# Supplementary material for: A phase I study of an adenoviral vector delivering a MUC1/CD40-ligand fusion protein in patients with advanced adenocarcinoma
Source: Nat Commun. 2022 Oct 28;13:6453. doi: 10.1038/s41467-022-33834-4 (PMC9616917; doi:10.1038/s41467-022-33834-4)
Supplement: Supplementary file 1 — Supplementary Information [file 41467_2022_33834_MOESM1_ESM.pdf]

Supplementary Information

A phase I study of an adenoviral vector delivering a MUC1/CD40-ligand fusion protein in patients with advanced adenocarcinoma

ID	Subtype	Primary	No. lines prior therapy	Concurrent endocrine therapy
A03	Breast	ER+HER2+	>3	Letrozole
A02	Breast	ER+HER2+	>3	None
A05	Ovarian	Serous	>3	-
A20	Lung	EGFR mutant	>3	-
A08	Breast	TNBC	>3	None
A07	Breast	ER+HER2-	>3	None
A10	Breast	ER+HER2-	>3	None
A13	Breast	ER+HER2-	>3	None
A12	Breast	ER-HER2+	>3	None
A01	Ovarian	Serous	3	-
A12	Breast	ER+HER2-	3	Exemestane
A19	Breast	ER+HER2-	2	None
A14	Ovarian	Endometroid	2	-
A04	Breast	ER+HER2-	1	Exemestane
A06	Breast	ER+HER2-	1	Letrozole
A11	Ovarian	Clear cell	1	-
A16	Ovarian	Serous	1	-
A21	Ovarian	Unknown	1	-
A17	Ovarian	Clear cell	1	-
A15	Breast	ER+HER2-	0	None
A09	Breast	ER+HER2-	0	Letrozole

Supplementary Table 1. Subject profile per primary tumour, subtype and number of lines of prior therapy. EGFR: epidermal growth factor receptor; ER: estrogen receptor; HER2: human epidermal growth factor receptor 2. Patient IDs were omitted for patient confidentiality.

System organ class<br><br>AE	Grade 3<br>N (%)	Grade 4/5<br>N (%)	Any Grade<br>N (%)
**All events**	2 (10%)	0	12 (57%)
**Blood and lymphatic system disorders**			
Anaemia	1 (5%)	0	1 (5%)
**General disorders and administration site conditions**			
Oedema limbs	1 (5%)	0	2 (10%)
Injection site reaction	0	0	15 (71%)
Fever	0	0	2 (10%)
Fatigue	0	0	2 (10%)
Pain	0	0	8 (38%)
**Gastrointestinal disorders**			
Bloating	0	0	1 (5%)
Diarrhoea	0	0	2 (10%)
Nausea	0	0	1 (5%)
Vomiting	0	0	1 (5%)
**Infections and infestations**			
Upper respiratory infection	0	0	2 (10%)
**Injury, poisoning and procedural complications**			
Dermatitis radiation	0	0	1 (5%)
**Investigations**			
ALT increase	0	0	1 (5%)
AST increase	1 (5%)	0	1 (5%)
Bilirubin increase	1 (5%)	0	2 (10%)
Creatinine increase	0	0	1 (5%)
**Metabolism and nutrition disorder**			
Anorexia	0	0	1 (5%)
Hyperkalaemia	0	0	1 (5%)
**Musculoskeletal and connective tissue disorder**			
Muscle cramps	0	0	1 (5%)
Nervous system disorders			
Headache	0	0	1 (5%)
**Respiratory, thoracic and mediastinal disorders**			
Cough	0	0	1 (5%)
**Skin and subcutaneous tissue disorders**			
Rash	0	0	2 (10%)
Psoriasis	0	0	1 (5%)

Supplementary Table 2. AE of any cause according to CTCAE v4.0. AE data have been aggregated after combining dose cohort groups. “All events” refer to total number of patients with AE of grades 3, 4 or 5 or any grade. Grade 3 events occurred in 2 out of 21 patients. There were no grades 4 or 5 AEs. One patient experienced grade 3 anaemia at month 3 and another experienced grade 3 elevated AST, blood bilirubin and peripheral oedema all of which were unrelated to study vaccine. AE, adverse event; ALT, alanine transaminase; AST, aspartate transaminase.

ID	Baseline measurable disease	Best % change from baseline	RECIST response
A03	Yes	-7.8947	SD
A01	No	-	NE
A04	Yes	12.5	SD
A06	No	-	NE
A10	Yes	5.7627	SD
A05	Yes	30.9524	PD
A12	Yes	NE[b]	PD
A19	Yes	104.1667	PD
A15	Yes	-2.9126	SD
A08	No	-	NE
A17	Yes	41.6667	PD
A18	Yes	1.4085	SD
A16[a]	Yes	-8	PD
A14	Yes	-25.8065	SD
A09	Yes	15	SD
A07	Yes	-4.5455	SD
A20[a]	No	new lesion	NE
A21	Yes	35.1648	PD
A13	Yes	6.8182	PD
A11	Yes	10.3448	SD
A02	Yes	3.7313	SD
		Total stable disease (RECIST)	10 (47.6%)
		Total progressive disease (RECIST)	7 (33.3%)

Supplementary Table 3. Summary of tumour response. [a] : Appearance of new lesion. [b] : New unmeasurable metastases. RECIST (v.1.1): Response Evaluation Criteria in Solid Tumours. SD: stable disease, PD: progressive disease. NE: non-evaluable.

**Peptide length:**	15	
**Offset:**	4	
**Overlap:**	11	
**Peptide count:**	8	
**Cleaned AA sequence:**	1	PDTRPAPGST APPAHGVTSA PDTRPAPGST APPAHGVTSA
**Index**	**Start AA**	**Peptide Sequence**
1	1	PDTRPAPGSTAPPAH
2	5	PAPGSTAPPAHGVTS
3	9	STAPPAHGVTSAPDT
4	13	PAHGVTSAPDTRPAP
5	17	VTSAPDTRPAPGSTA
6[a]	21	PDTRPAPGSTAPPAH
7[b]	25	PAPGSTAPPAHGVTS
8[c]	29	APGSTAPPAHGVTSA

Supplementary Table 4: MUC1 peptides used for immunological assays. [a]: identical to 1. [b]: identical to 2. [c]: end of sequence, with a 14 amino acid overlap.

Panel 1

Metal	Antibody	Clone	Vendor	Catalog	Working Conc (µg/ml)
**89**	Barcode (CD45)	HI30	Fluidigm	3089003B	7.5
**106, 113, 115**	Barcode (CD45)	HI30	Biolegend	304002	5
**110**	CD19	HIB19	Biolegend	302202	6
**111**	CD45RA	HI100	Biolegend	304102	5
**112**	CD14	TüK4	Invitrogen	MA5-16956	5
**116**	CD8	SK1	Biolegend	344702	5
**139**	HLA-DR	L243	Biolegend	307602	6
**141**	4-1BB (CD137)	4B4-1	Biolegend	309802	8
**142**	CD45RO	UCHL1	Biolegend	304202	5
**143**	CD3	UCHT1	Biolegend	300402	5
**144**	CD40L (CD154)	24-31	Biolegend	310835	8
**145**	IL-4	8D4-8	Biolegend	500702	3
**146**	Anti-FITC (TCRγδ)	FIT-22	Biolegend	408302	6
**147**	PD-1	EH12.2H7	Biolegend	329902	8
**148**	CD4	SK3	Biolegend	344602	6
**149**	CD127	A019D5	Biolegend	351337	6
**150**	IL-22	22URTI	Fluidigm	3150007B	5
**151**	GATA3	TWAJ	eBioscience	14-9966-82	3
**152**	TNF-α	Mab11	Biolegend	502902	2
**153**	CD25	2A3	BD Pharmingen	340739	5
**154**	CD7	CD7-6B7	Biolegend	343102	5
**155**	CD152	BNI3	BD Pharmingen	555850	3
**156**	CD28	CD28.2	Biolegend	302923	8
**157**	CXCR5	RF8B2	BD Pharmingen	552032	5
**158**	CCR7	G043H7	Biolegend	353202	5
**159**	LAG-3	11C3C65	Biolegend	369302	8
**160**	CD161	HP-3G10	Biolegend	339902	6
**161**	CD56	NCAM16.2	BD Pharmingen	559043	5
**162**	RORgt	AFKJS-9	eBioscience	14-6988-82	5
**163**	TBX21/Tbet	4B10	BioXell	BE0100	5
**164**	Anti-PE(TCRαβ)	PE001	Biolegend	408102	6
**165**	FoxP3	236A/E7	eBioscience	14-4777-82	5
**166**	Ki67	20Raj1	eBioscience	14-5699-82	4
**168**	IFN-γ	B27	Biolegend	506502	4
**169**	IL17A	BL168	Biolegend	512302	3
**170**	IL-8	BH0814	Biolegend	514602	3
**171**	TIGIT	MBSA43	Invitrogen	16-9500-82	5
**172**	CD31	WM59	Biolegend	303102	6
**173**	GranzymeB	CLB-GB11	Abcam	ab103159	4
**174**	CD69	FN50	Biolegend	310902	5
**175**	IL-10	JES3-19F1	Biolegend	501402	5
**176**	ICOS	C398.4A	Biolegend	313502	8
**209**	CD16	3G8	Fluidigm	3209002B	5
**Primary Antibody**	**Antibody**	**Clone**	**Vendor**		**Dilution**
	PE anti-TCRαβ	T10B9.1A-31	BD Pharmingen	555548	1:2.5
	FITC anti-TCRγδ	5A6.E9	Invitrogen	MHGD01	1:20

Supplementary Table 5. Mass cytometry panel 1, T cell focused (P1).

Panel 2

Metal	Antibody	Clone	Vendor	Catalog	Working Conc (μg/ml)
**89**	Barcode (CD45)	HI30	Fluidigm	3089003B	7.5
**106, 113, 115**	Barcode (CD45)	HI30	Biolegend	304002	5
**110**	CD19	HIB19	Biolegend	302202	6
**111**	CD45RA	HI100	Biolegend	304102	5
**112**	CD14	TüK4	Invitrogen	MA5-16956	5
**116**	CD8	SK1	Biolegend	344702	5
**139**	HLA-DR	L243	Biolegend	307602	6
**141**	ICOS-L (CD275)	2D3	Biolegend	309402	8
**142**	CD11B	ICRF44	Biolegend	301302	6
**143**	CD3	UCHT1	Biolegend	300402	5
**144**	CD206	15-2	Biolegend	321127	8
**145**	CD10	HI10a	Biolegend	312202	7
**146**	IgD	Ia6-2	Biolegend	348202	6
**147**	CD1C	L161	Biolegend	331502	7
**148**	CD4	SK3	Biolegend	344602	6
**149**	CD21	Bu32	Biolegend	354902	5
**150**	CD273 (PD-L2)	MIH18	Biolegend	345502	7
**151**	CXCR4	12G5	Biolegend	306502	5
**152**	NKp46	9E2	Biolegend	331902	5
**153**	CD303	201A	Biolegend	354202	6
**154**	CD27	O323	Biolegend	302802	6
**155**	IgG	G18-145	BD Pharmingen	555784	4
**156**	PD-L1	29E.2A3	Biolegend	329719	6
**157**	CD11c	Bu15	Biolegend	337202	3
**158**	CD141	M80	Biolegend	344102	6
**159**	B7-H4	MIH43	Biolegend	358102	8
**160**	IgM	MHM-88	Biolegend	314502	5
**161**	CD56	NCAM16.2	BD Pharmingen	559043	5
**162**	CD62L	DREG-56	Biolegend	304835	5
**163**	CD38	HIT2	Biolegend	303502	5
**164**	CX3CR1	K0124E1	Biolegend	355702	6
**165**	CCR7	G043H7	Biolegend	353202	5
**166**	CD24	ML5	Biolegend	311102	6
**167**	CD80	2D10	Biolegend	305202	5
**168**	IgA	G18-1	BD Pharmingen	555886	4
**169**	CD304	12C2	Biolegend	354502	6
**170**	NKG2D	1D11	Biolegend	320802	5
**171**	CD23 (FcεRII)	EBVCS-5	Biolegend	338502	6
**172**	CD86	IT2.2	Biolegend	305402	8
**173**	Granzyme B	CLB-GB11	Abcam	ab103159	4
**174**	CD40	5C3	Biolegend	334302	5
**175**	IL-10	JES3-19F1	Biolegend	501402	5
**176**	CD163	RM3/1	Biolegend	326502	8
**209**	CD16	3G8	Fluidigm	3209002B	6

Supplementary Table 6. Mass cytometry panel 2, with a B cell/APC focus (P2).

pre-vaccination (n=10)

ID	RECIST	Cancer Type
A16	PD	Ovarian
A15	SD	Breast
A05	PD	Ovarian
A07	SD	Breast
A20	NE (new lesion)	Lung
A11	SD	Ovarian
A13	PD	Ovarian
A06	NE (no progression)	Breast
A04	SD	Breast
A18	SD	Breast

post-vaccination (n=10)

ID	RECIST	Cancer Type
A05	PD	Ovarian
A20	NE (new lesion)	Lung
A11	SD	Ovarian
A06	NE (no progression)	Breast
A10	SD	Breast
A17	PD	Ovarian
A09	SD	Breast
A02	SD	Breast
A12	PD	Breast
A04	SD	Breast

Supplementary Table 7. Cancer samples used in the network graphs in Figure 4. Cancer samples used in pre and post-vaccination immune networks, their response classification according to Response Evaluation Criteria in Solid Tumours (RECIST v. 1.1) and cancer type are listed. PD: progressive disease, SD: stable disease, NE: non-evaluable (due to lack of baseline scan).

network property	description	pre-vaccination	post-vaccination
graph density	proportion of all possible connections that are actual connections	0.114	0.113
degree of centralisation	measure of how connected a given node is to others	0.114	0.128
average path length	average number of steps along shortest paths for all pairs of nodes (efficiency of information transfer)	2.731	2.726
modularity	structural measure; high modularity indicates dense connections between nodes in modules but sparse connections outside of modules	0.118	0.104
negative edges	negatively correlated connections	101	76
positive edges	positively correlated connections	260	281

Supplementary Table 8. Network properties of the network graphs in Figure 4. Network properties are described and tabulated for immune network maps of cancer patients pre and post-vaccination. Network properties were calculated using the igraph package in R.

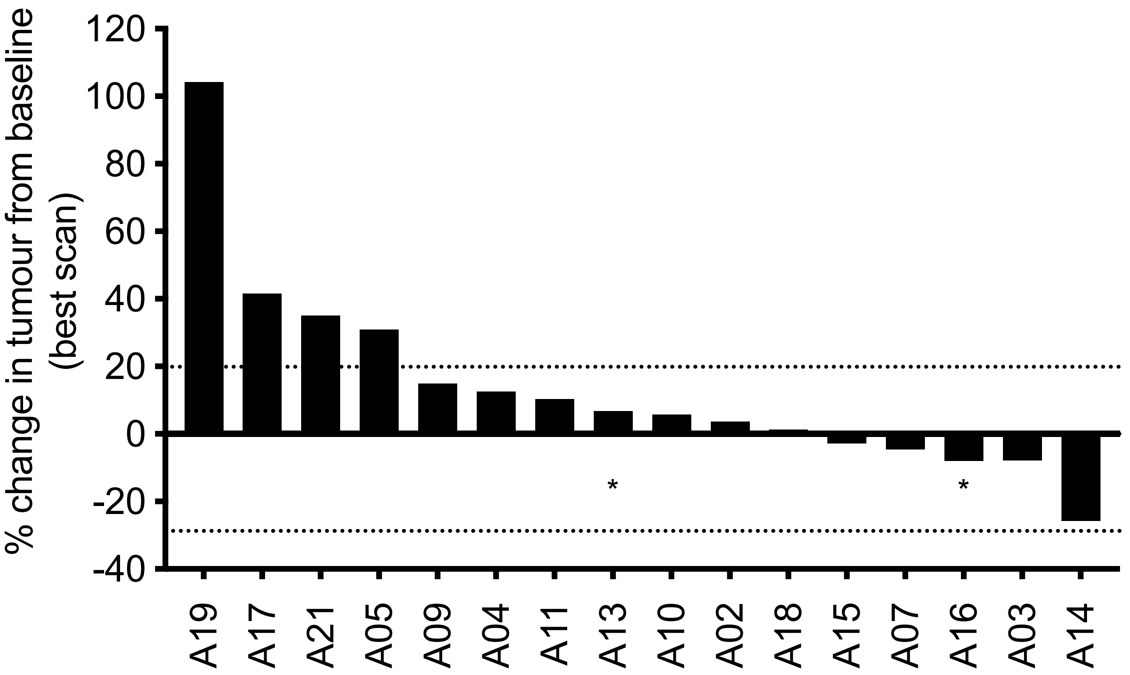

Supplementary Figure 1. Clinical response to vaccination. Percentage change in tumour measurement (of best scan) from pre-treatment for patients with measurable disease, taken two months after last vaccination for dose levels 1-6 and on days 52, 117 and 172 for dose level 7. Dotted line indicates cut-off for RECIST (v. 1.1) criteria (progressive disease, PD: at least 20% increase, partial response: at least 30% decrease, stable disease, SD: neither sufficient shrinkage for partial response or sufficient increase for progression). Patients A01, A06, A08, A12 and A20 were excluded from this graph due to non-evaluable disease (A01, A06, A20) or non measurable metastatic disease at second scan (A12), while A08 did not have a follow-up scan. *: presented with additional lesions and therefore considered PD.

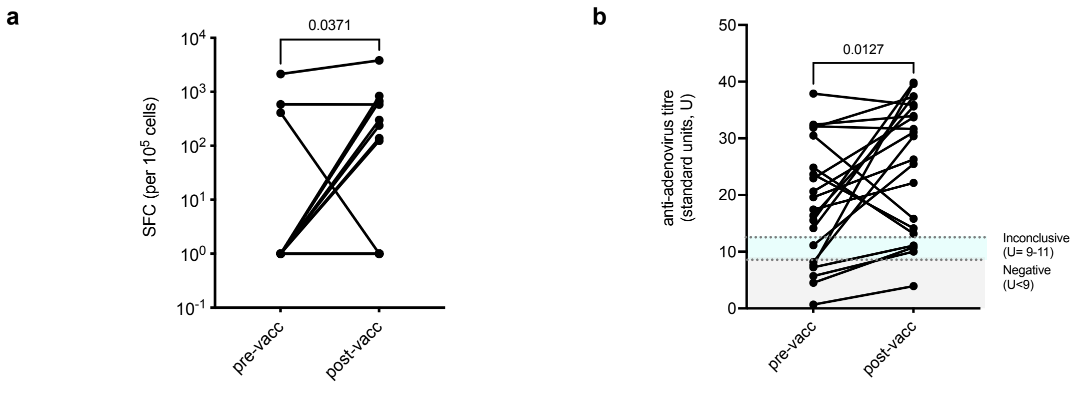

Supplementary Figure 2. Serology of MUC1 response. (a) MUC1-specific T cells were detected by IFN-γ ELISpot assay on serially collected PBMC samples pre and post-vaccination. Results are represented as spot-forming cells (SFC)/10^5 PBMCs. Background values were defined as the mean numbers of the negative controls and deducted from the mean numbers obtained with the MUC1 peptide pool. Mean spot counts of >2 x background values were considered positive. (b) Plasma anti-adenovirus antibody titres were determined by ELISA. Each data point represents the mean value of triplicate readouts. Standard Unit values > 11 were considered positive, <9 were considered negative, and 9-11 were considered inconclusive. p values were determined by two-tailed Wilcoxon matched-pairs signed rank test. n=21 for both panels.

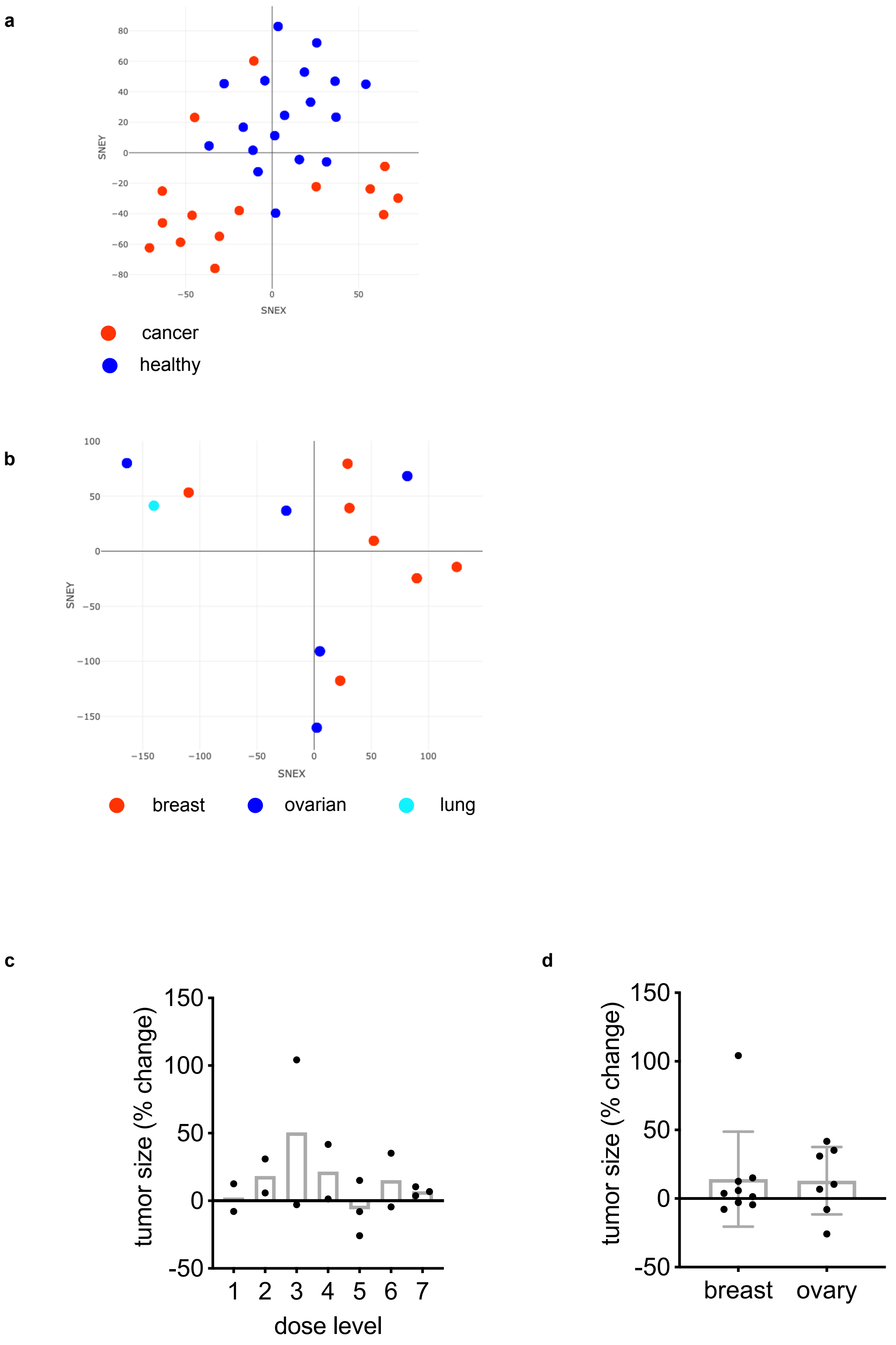

Supplementary Figure 3. Heterogeneity in PBMC and tumour data. (a) Clustering of healthy (blue) vs. pre-vaccination cancer (red) patients by tSNE (n=33), using P1 data.(b) Clustering of mass cytometry data from baseline PBMC samples from cancer patients by type of adenocarcinoma, using P2 data (n=13). Some samples were excluded due to insufficient cell numbers (c,d) Percent change in tumour size from baseline stratified by (c) dose level (dose levels 1, 2, 3, 4, 6, n=2, dose levels 5, 7, n=3) or (d) cancer type, excluding subjects with non-detectable disease at baseline (breast, n=9, ovary, n=7). Error bars indicate SE of mean.

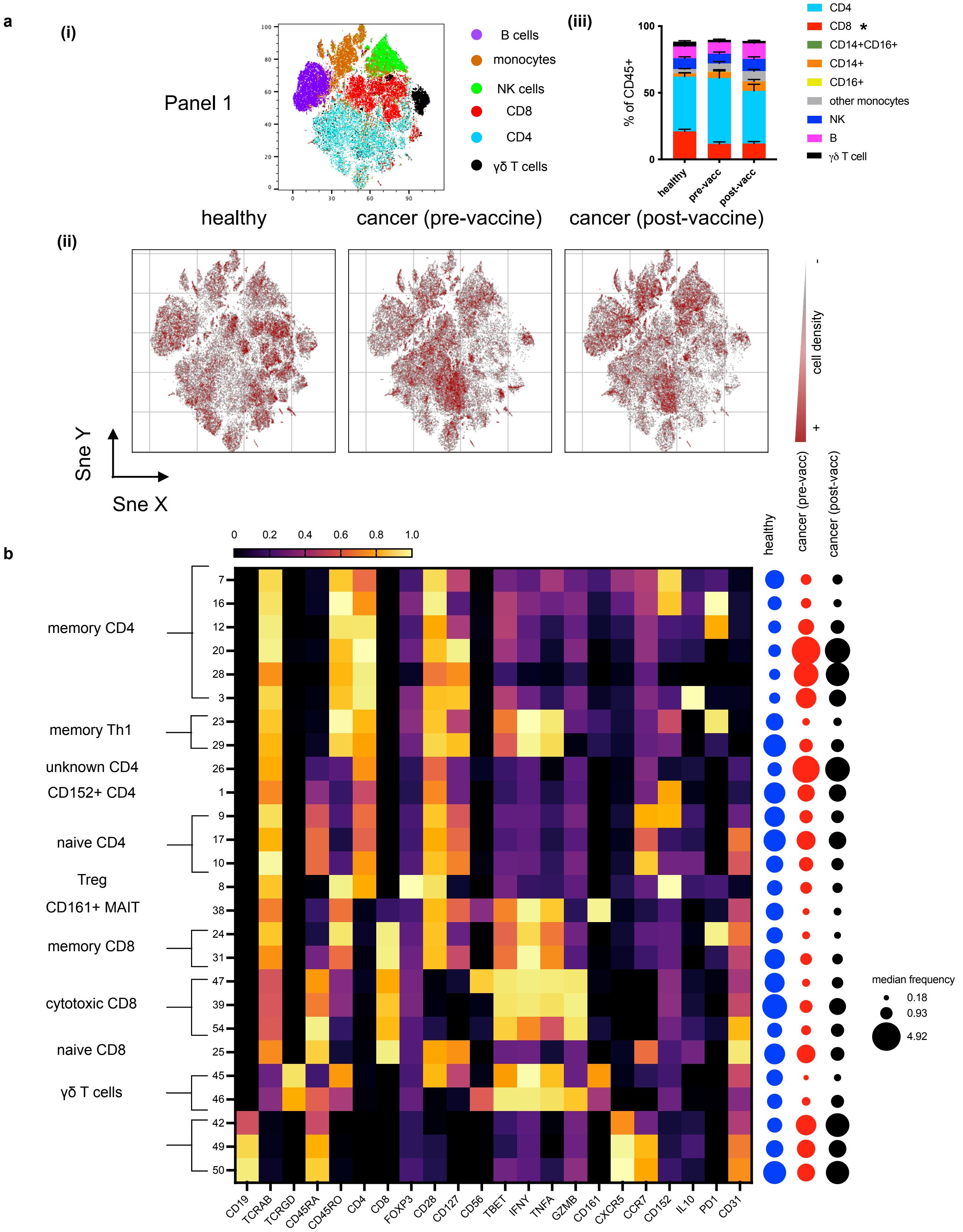

Supplementary Figure 4. Aberrant immunome architecture in cancer as assayed by a T-cell focused panel. Mass cytometry data from PBMCs of healthy donors and cancer patients pre and post-vaccination was analysed. (a) Using panel 1 (P1) data, (i) major immune lineages were gated and overlaid on a tSNE map of all cells (sampled from all samples) as a reference for (ii) cell density embedding of healthy, pre and post-vaccination cancer patients, where red areas represent higher cell densities. (iii) Major immune lineages were then manually gated and compared across groups *: significantly different ($p=0.0007$) between healthy and pre-vaccination cancer by Tukey's multiple comparisons test. (b) After FlowSOM clustering, significantly different ($p<0.05$) clusters between healthy (blue) and cancer pre-vaccination (red) were identified using Mann-Whitney U test (two-tailed) of cluster frequencies. The phenotypes of these clusters are summarised in heatmaps in context with cancer post-vaccination (black). Some samples were excluded from unsupervised analyses due to quality control cutoffs. Some samples were excluded from supervised analyses due to insufficient cell numbers. The median frequency of each cluster was represented by the size of dots. Heatmaps represent scaled median arcsin expression of each marker. Source data are provided as a Source Data file. Unsupervised analyses in (a)(i), (b): healthy (n=18), pre (n=10) and post-vaccination (n=12) Supervised analyses in (a)(ii): healthy (n=18), pre (n=12) and post-vaccination (n=13).

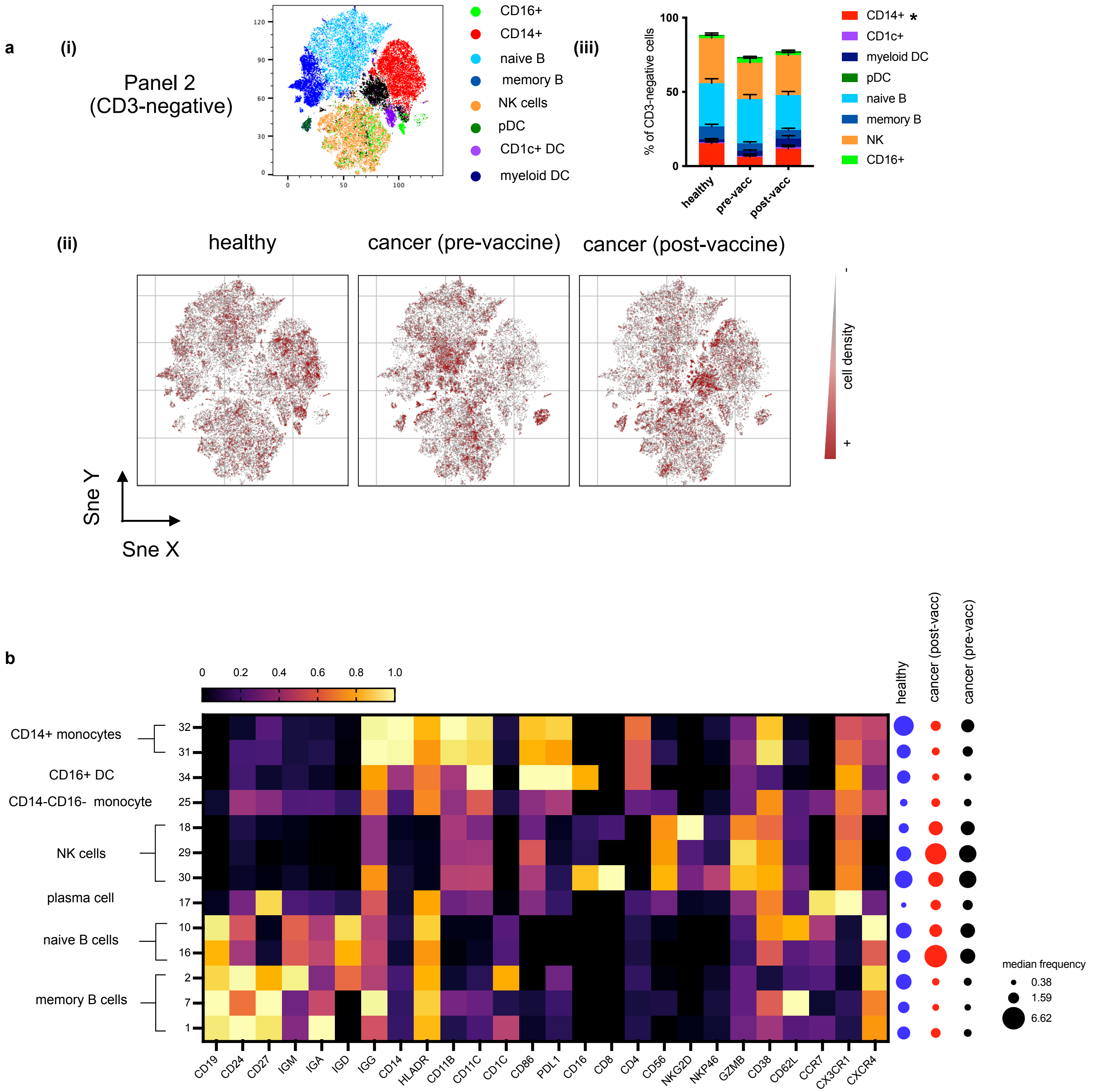

Supplementary Figure 5. Aberrant immunome as assayed by an APC/B cell focused panel. Mass cytometry data from PBMCs of healthy donors and cancer patients pre and post-vaccination was analysed. (a) Using panel 2 (P2) data gated on CD3-negative cells, (i) major immune lineages were gated and overlaid on a tSNE map of all cells as a reference for (ii) cell density embedding of healthy, pre and post-vaccination cancer patients, where red areas represent higher cell densities. (iii) Major immune lineages were then manually gated and compared across groups. *: significantly different (p=0.0171) between healthy and pre-vaccination cancer by Tukey's multiple comparisons test. (b) After FlowSOM clustering, significantly different (p<0.05) clusters between healthy (blue) and cancer pre-vaccination (red) were identified using Mann-Whitney U test (two-tailed) of cluster frequencies. The phenotypes of these clusters are summarised in heatmaps in context with cancer post-vaccination (black). The median frequency of each cluster was represented by the size of dots. Some samples were excluded from unsupervised analyses due to quality control cutoffs. Some samples were excluded from supervised analyses due to insufficient cell numbers. Heatmaps represent scaled median arcsin expression of each marker. Source data are provided as a Source Data file. Unsupervised analyses in (a)(i), (b): healthy (n=18), pre (n=13) and post-vaccination (n=13) Supervised analyses in (a)(ii): healthy (n=18), pre (n=18) and post-vaccination (n=17).

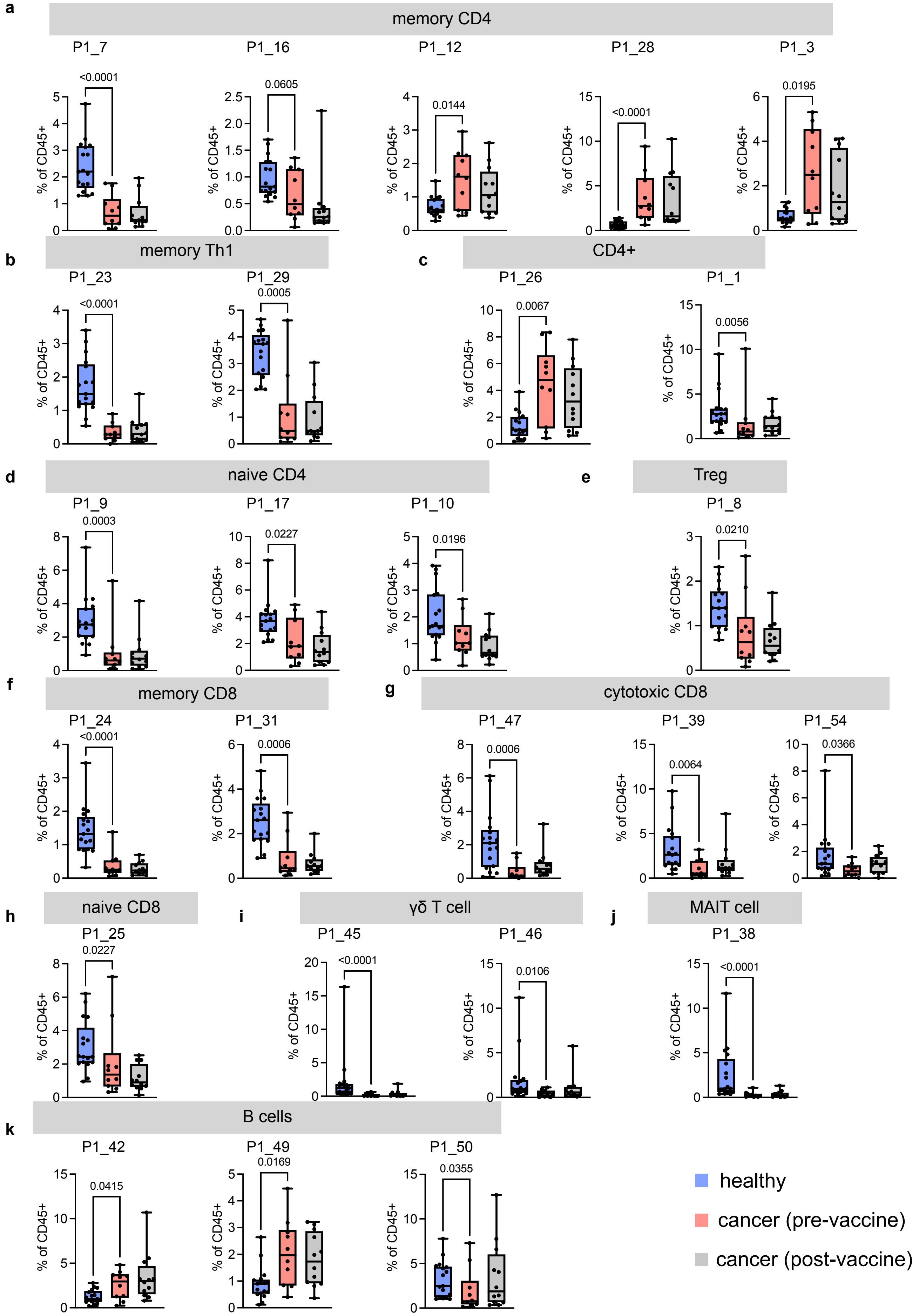

Supplementary Figure 6. Differences in healthy vs. cancer using a T cell focused panel (P1). PBMCs from healthy (blue, n=17) volunteers and cancer patients pre (red, n=10) and post-vaccination (grey, n=12) were analysed by mass cytometry using a T cell focused panel. All p values shown are comparisons between healthy and pre-vaccine as determined by Mann Whitney U test (two-tailed). Clusters were grouped according to phenotype, (a) memory CD4, (b) memory Th1, (c) other CD4, (d) naive CD4, (e) Treg, (f) memory CD8, (g) cytotoxic CD8, (h) naive CD8, (i)γδ T, (j) MAIT, (k) B cells. Box and whiskers plot represent median and minimum/maximum values determined by unsupervised analysis of the data. Some samples were excluded from unsupervised analyses for not meeting quality control cutoffs (see Methods). Source data are provided as a Source Data file.

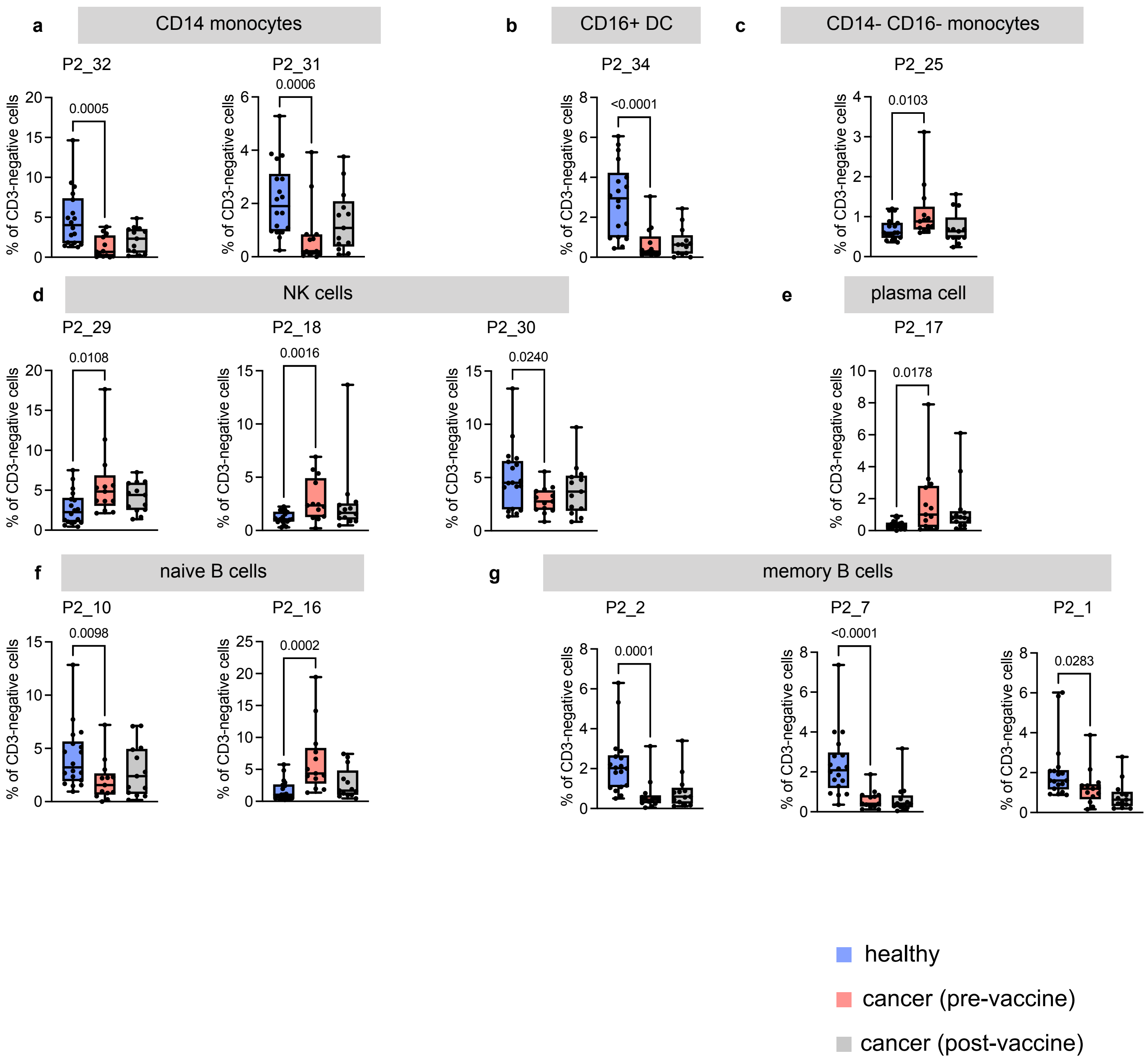

Supplementary Figure 7. Differences in healthy vs. cancer using a B cell/APC focused panel (P2). PBMCs from healthy (blue, n=18) volunteers and cancer patients pre (red, n=13) and post-vaccination (grey, n=13) were analysed by mass cytometry using a B cell/APC focused panel. All p values shown are comparisons between healthy and pre-vaccine as determined by Mann Whitney U test (two-tailed). Clusters were grouped according to phenotype: (a) CD14+ monocytes, (b) CD16+ DC, (c) CD14- CD16- monocytes, (d) NK cells, (e) plasma cells, (f) naive B cells, and (g) memory B cells. Box and whiskers plot represent median and minimum/maximum values determined by unsupervised analysis of the data. T Some samples were excluded from unsupervised analyses for not meeting quality control cutoffs (see Methods). Source data are provided as a Source Data file.

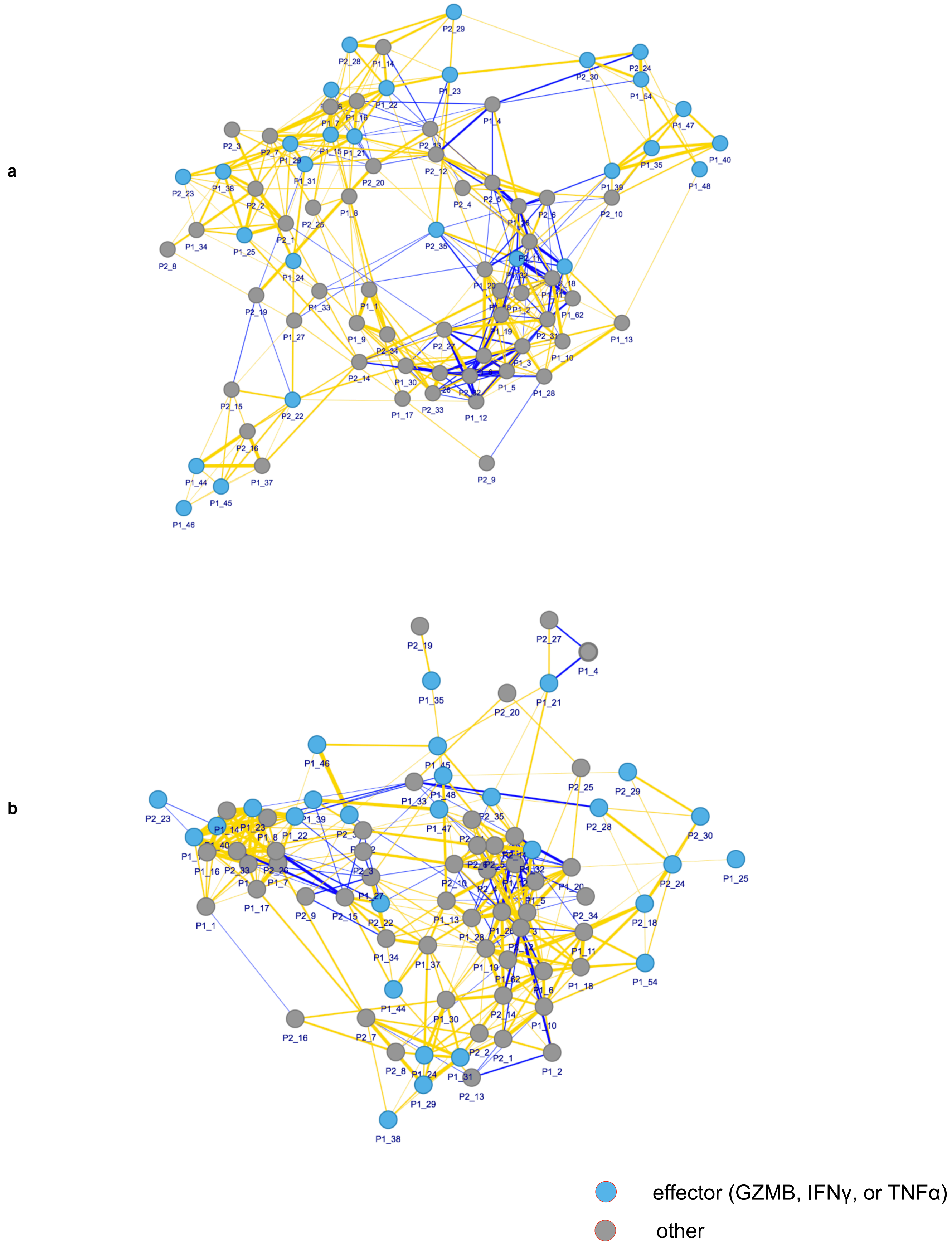

Supplementary Figure 8. Vaccination reshapes effector cell networks in cancer patients. Network analysis of mass cytometry data in n=10 patients (a) pre and (b) post-vaccination (correlation coefficient: 0.6). Yellow edges represent negative correlations and blue edges represent positive correlations. Nodes are coloured by phenotype (blue: GZMB, IFNγ, or TNFα expression , grey: other). The thickness of the line represents the strength of the correlation. GZMB: granzyme B.

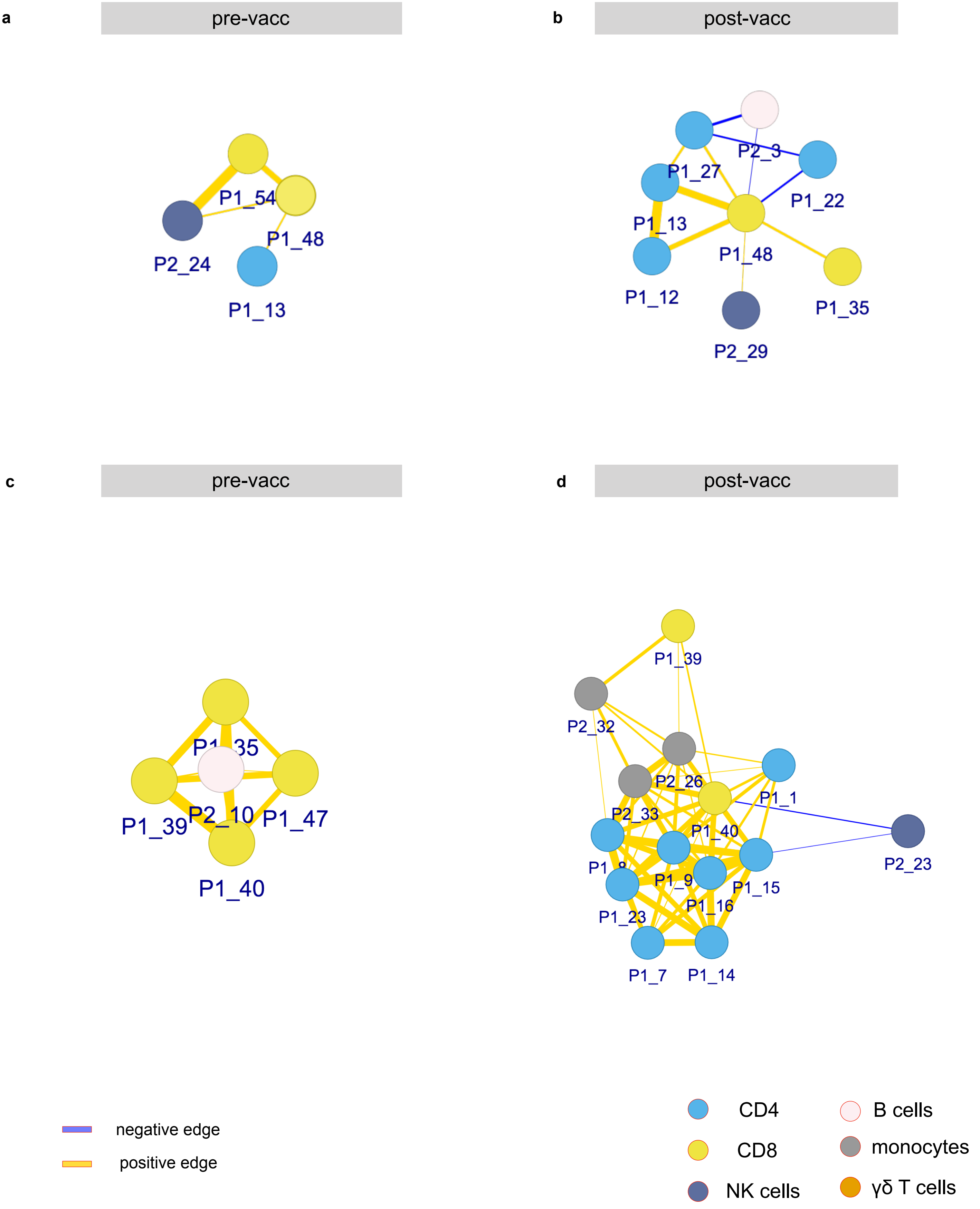

Supplementary Figure 9. Vaccination partially restores network complexity. Network analysis of mass cytometry data in n=10 patients pre (a, c) and (b, d) post-vaccination (correlation coefficient: 0.6) as in Figure 4. The nodes (a-b) P1_48 and (c-d) P1_40 in each group are displayed with their most immediate connections. Nodes are coloured by cell type (blue: CD4, yellow: CD8, beige: B cells, indigo: NK cells, grey: monocytes, orange: γδ T cells). Yellow edges represent negative correlations and blue edges represent positive correlations. The thickness of the line represents the strength of the correlation.

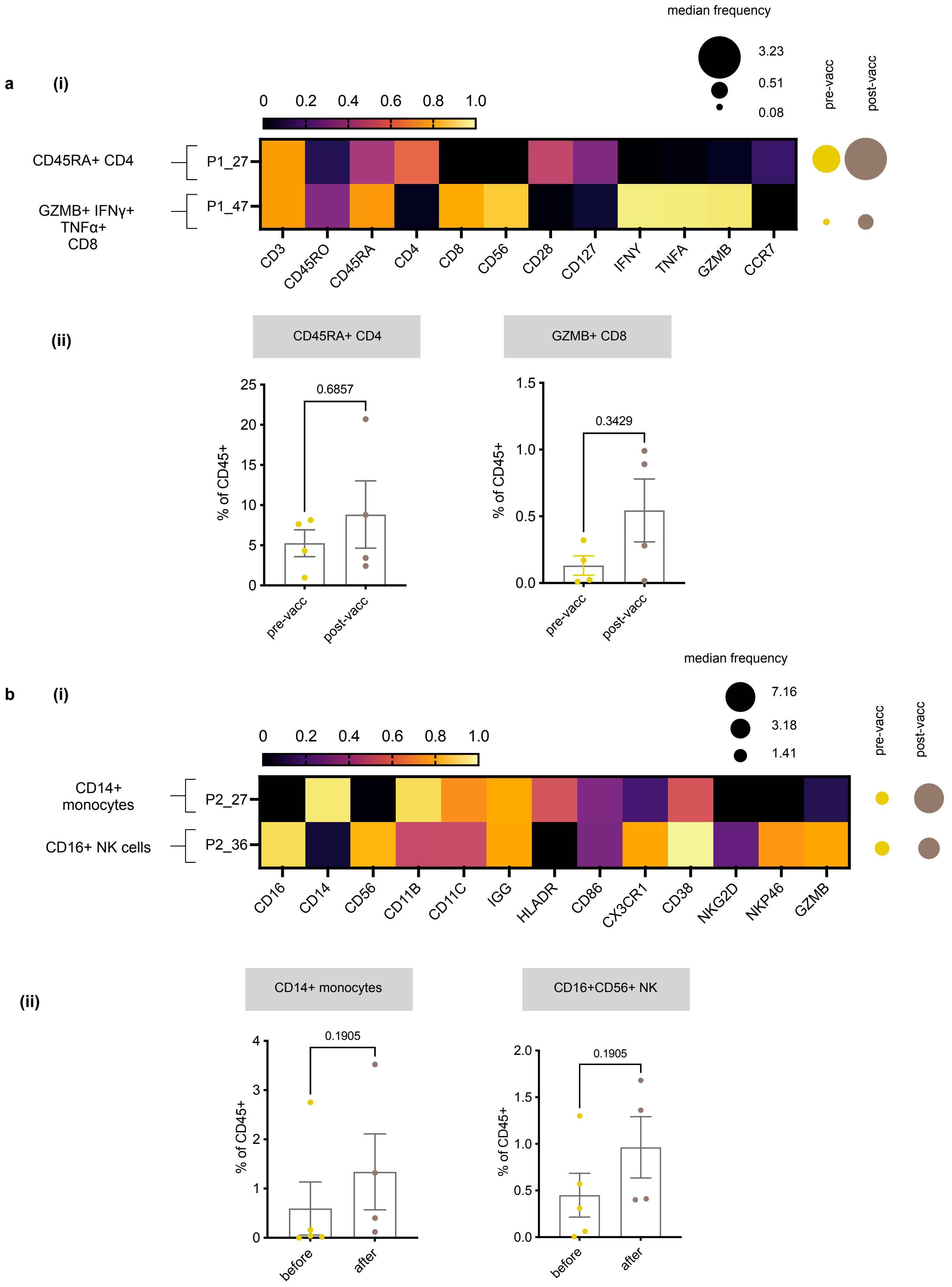

Supplementary Figure 10. Vaccination effects in patients with progressive disease (PD). Mass cytometry data was clustered by FlowSOM and analysed for clusters that differed pre (yellow circles) and post-vaccination (brown circles) in patients with PD as defined by RECIST v1.1, using (a) Panel 1 (n=4) and (b) Panel 2 (n=5 pre-vaccination, n=4 post-vaccination). (a(i), b(i)) The phenotypes of these clusters are summarised in the heatmaps of marker expression. The median frequency of each cluster was represented by the size of dots.(a(ii), b(ii)) Manual gating validation of selected subsets. Heatmap represents the scaled arcsin median expression of each marker. Mann-Whitney U tests (two-tailed) were performed to compare between groups. Error bars indicate SE of mean. Some samples were excluded from supervised analyses due to insufficient cell numbers. Source data are provided as a Source Data file.

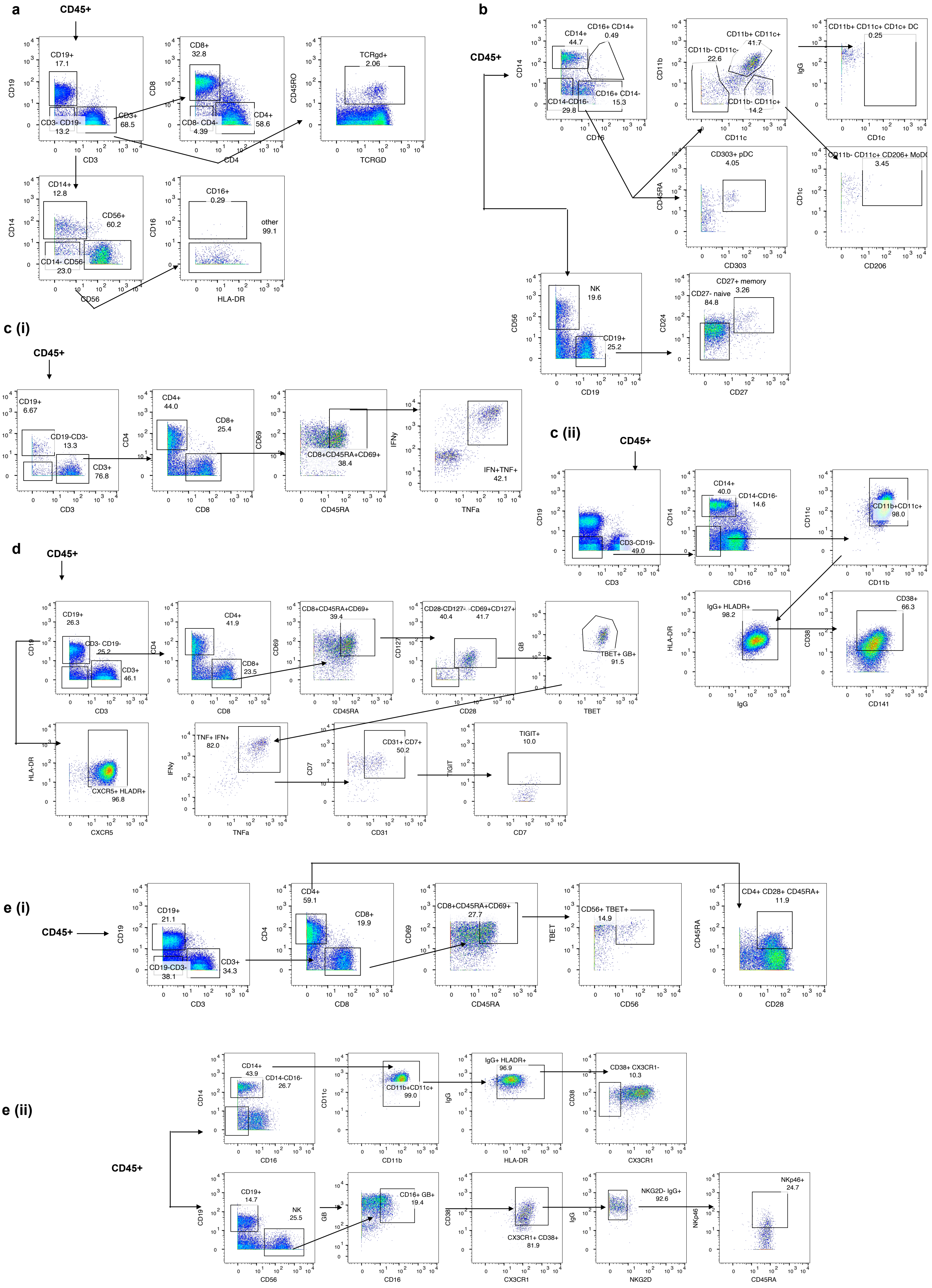

Supplementary Figure 11. Gating strategy for manual gating. Gating strategy for (a) Supplementary Figure 4a(iii), (b) Supplementary Figure 5a(iii), (c) (i) Figure 2b, (ii) Figure 2d, (d) Figure 4b, (e) (i) Supplementary Figure 10a(ii) , (e)(ii) Supplementary Figure 10b(ii).

Supplementary Notes
Study Protocol

Title: Ad-sig-hMUC-1/ecdCD40L vector vaccine for immunotherapy of epithelial cancers.

Department of Medical Oncology &
Division of Cellular and Molecular Research

National Cancer Centre Singapore

<u>Sponsored by :</u>

Singapore Clinical Research Institute (SCRI)

Trial Management Group

**Principal Investigator:**<br>Dr Toh Han Chong	National Cancer Center, Singapore
**Co-Investigators:**<br>Dr John Chia<br>Dr Tan Eng Huat<br>Dr Darren Lim<br>Dr Ang Mei Kim<br>Dr Toh Chee Keong<br>Dr Choo Su Pin<br>Dr Yap Yoon Sim<br>Dr Ng Quan Sing<br>Dr Daniel Tan Shao Weng<br>Dr David Tai Wai Meng<br>Dr Matthew Ng Chau Hsien<br>Dr Ravindran Kanesvaran<br>Dr Amit Jain<br>Dr Tira Tan Jing Ying<br>Dr Elaine Lim<br>Dr Rebecca Dent<br>Dr Amanda Seet Oon Lim<br>Dr Jack Chan Junjie<br>Dr Joycelyn Lee Jie Xin<br>Dr Lim Chiew Woon<br>Dr Esther Chang Wei Yin<br>Dr Gillianne Lai Geet Yi<br>Dr Huren Sivaraj<br>Dr Chay Wen Yee	National Cancer Center, Singapore
**Translational Science Collaborator**<br>Deisseroth Albert<br>John Connollly	Institute of Molecular and Cellular Biology, Singapore
**IP Management**<br>Peter Wang<br>Rachael Cheong<br>Cheryl Chiang<br>Koh Lip Seng	National Cancer Center, Singapore
**Statistician**<br>Tan Sze Huey	National Cancer Center, Singapore
**Study Coordinator**<br>Chong Hui Shan	National Cancer Center, Singapore

Contents

1.INTRODUCTION

2.SUMMARY

3.OBJECTIVES AND ENDPOINTS
3.1.Primary Objective
3.2.Secondary Objective

4.BACKGROUND AND RATIONALE
4.1.MUC-1 in Cancers of the Breast, Colon, Ovary, Prostate and Lung
4.2.Clinical Experience with CD40L Vaccines and Antibodies to the CD40 Receptor
4.3.Description of Ad-sig-ecd-hMUC-1/ecdCD40L Vaccine
4.4 Relevant Pre-Clinical Studies

5.STUDY DESIGN
5.1.Type of Study
5.2.Treatment Plan
5.3.Adsig-hMUC-1/ecdCD40L Vector Administration
5.4.Primary Objective
5.5. Secondary Objectives: Determination of the Optimal Biological Dose
5.6.Expected Number of Subjects

6.STATISTICAL ANALYSIS
6.1.Toxicity Evaluation

7.PATIENT SELECTION
7.1.Inclusion Criteria
7.2.Exclusion Criteria

8.SCREENING EVALUATION, COUNSELLING, PATIENT CONSENT
8.1.Screening of Patients
8.2.Patient Counselling
8.3.Cost Considerations
8.4.Patient Consent

9.TREATMENT PLAN
9.1.Timing of Enrollment
9.2.Preparation of Vaccine for Injection in the Clinic
9.3.Vaccine Dose and Schedule

10.DRUG SUPPLIES AND PREPARATION
10.1Vector Production (redacted)
10.2Viral Vector Particle Supply (redacted)
10.3Viral Vector Dilution (redacted)
10.4SOP for Handling Vector Vaccine Preparation Spill

11.SUBJECT SAFETY
11.1 Adenoviral Vaccine Toxicities
11.2 Known Toxicity of CD40 Ligand
11.3 Potential Ad-sig-hMUC-1/ecdCD40L Vector Specific Adverse Events
11.4 Patient Protection
11.5 Severe Adverse Events Definition
11.6 Serious Adverse Event Reporting
11.7 Adverse Event Definition
11.8 Reporting Non-SAE Adverse Events
11.9 Toxicity Grading
11.10.Criteria for Removal From Study

12.PATIENT EVALUATION
12.1 Study Calendar
12.2 Baseline / Pre-immunisation
12.3 Pre-vaccination
12.4 Post-vaccination – 24 hrs and 48 hrs
12.5 Post Vaccine Evaluation – Day 15 (cohorts 1-6)
12.6 Post Vaccine Evaluation – Month 1,2, 3 (cohorts 1-6)
12.7 Post Vaccine Evaluation - Monthly Follow-up visits (cohort 7)

13.RESPONSE EVALUATION

14.STUDY STOPPING RULES

15.DATA MANAGEMENT
15.1.Data Management Team (redacted)
15.2.Study Documentation (redacted)
15.3.Monitoring and Audit (redacted)
15.4.Data Entry (redacted)
15.5.Data Archive (redacted)

16.ETHICAL AND REGULATOR STANDARDS
16.1.Ethical Principles
16.2.Patient Confidentiality
16.3.Withdrawal of Patients

17. REFERENCES

1. INTRODUCTION

Cancer of the breast, lung, colon, prostate or ovary remains a highly significant cause of morbidity and mortality worldwide.[1] The vast majority of deaths in epithelial cancer are due to metastatic disease.

Chemotherapy (either sequential single agents or combination) and endocrine therapy remain the standard treatment for metastatic breast cancer as they have demonstrated activity in this setting. However, even with these therapies, median survival remains approximately two years, and less than 3% of patients will achieve long-term survival.[2] Recently, therapies targeted to the Her2/neu receptor, such as chemotherapy with trastuzumab, a humanized monoclonal antibody targeting the Her2/neu protein, or lapatinib with capecitabine, or lapatinib with an aromatase inhibitor, have been shown to improve survival in patients with metastatic breast cancer.[3] Targeted therapies are also being developed for cancers of the ovary, prostate, colon and lung. While these novel, targeted therapies have had an enormous positive impact on the lives of some patients, there remains a large population of patients with metastatic cancer who would benefit from a novel, less toxic therapy.

One potentially promising avenue for the development of new anti-cancer agents is stimulating the immune system to recognize and eradicate cancer cells. This protocol proposes to study the toxicity and immune system effects of a vector-based anti-cancer vaccine as treatment for men or women with metastatic epithelial cancer who have recurrent or metastatic carcinoma of the breast, ovary, colon, prostate or lung.

The notion that the immune system could be used to generate an anti-tumor response was first explored by William Coley in the 1890s with the development of a vaccine comprised of attenuated *Streptococcus pyogenes* and *Serratia marcescens* that would stimulate the immune system to non-specifically react against tumor.[4] Several hundred cancer patients were injected with “Coley’s toxins” and of these, a significant number achieved tumor regression and even cure. However, with the advent of radiation and chemotherapy, cancer immunotherapy was all but forgotten until the latter half of the twentieth century when scientists began re-exploring methods to exploit the host’s own immune system to specifically destroy malignant cells.

Numerous cancer vaccines have been developed and tested with the most promising results seen in lymphoma and hormone-refractory prostate cancer patients.[5-6] Unfortunately, the cumulative objective response rate for solid tumor vaccines has been only 3.8%. This lack of responsiveness of the immune system toward cancer cells can be at least partly explained by tolerance, which develops because cancer cells are covered by “self antigens” that have been present on normal cells from birth. Although these self antigens may be altered in cancer (through their level of expression, the acquisition of somatic mutations, or the alteration of the pattern of glycosylation on the protein backbone), the immune response is minimal since the altered antigens do not come into contact with the antigen presenting cells [e.g. dendritic cells, (DCs)]. Furthermore, T cells, once activated against the cancer associated antigens, do not home to extravascular sites unless there is inflammation or a danger signal in that tissue. Thus, an ideal tumor vaccine would both target a uniquely expressed antigen on tumor cells and overcome the immune system’s tolerance to that antigen.

This is a single-site phase I non-randomized open label dose escalation trial of the Ad-sig-hMUC-1 ecdCD40L vector cancer vaccine (MUC-1 vector vaccine) for men or women with metastatic or recurrent disease who have been on their current therapy or on no therapy for a one month period prior to enrolling in this trial, and who have had an elevated serum MUC-1 level at some point since their initial diagnosis of epithelial cancer. Subsequent to signature of informed consent, eligible subjects with measurable or non-measurable metastatic epithelial cancer will receive one subcutaneous injection of MUC-1 vaccine in dose cohorts 1-4 (study part one). It is expected to enroll and treat a minimum of 6 and maximum of 24 patients for study part one. The emphasis of study part one is safety and tolerability of one vector injection. If the objective of safety is met in study part one and a tolerable dose level is defined, Expansion Cohorts 5 and 6 will be added as study part two. In study part two, the eligible patients will receive two subcutaneous injections of the MUC-1 vaccine (7 days apart) in dose cohort 5, and three subcutaneous injections of the MUC-1 vaccine on days 1, 8 and 22 in cohort

6. In dose cohorts 5 and 6, the dose of the vaccine will be the highest among cohorts 1-4 that was tolerated. It's expected to enroll and treat a minimum of 3 and maximum of 24 patients for study part two. Serum MUC-1 levels and changes in the function of the immune system will also be evaluated.

In study part three (which will be referred to hereafter as Cohort 7), the eligible patients will receive 5 subcutaneous injections of the MUC-1 vaccine on Days 1, 8, 22, 52 and 82. This schedule of injections will be administered to six individual patients. Serum MUC-1 levels and changes in the function of the immune system will be evaluated prior to the administration of each of the vaccinations. In addition, CT scans will be carried out prior to vaccinations 4 at 30 days and 90 days after the last injections. The goal of Part 3 is to determine the safety and efficacy of 5 vaccinations.

2. SUMMARY

Design	Phase I non randomized, single institution open label study.
Drugs	Subcutaneous injection of the Ad-sig-hMUC-1/ecdCD40L adenoviral vaccine
Objectives	1) To determine the MTD or the optimal biological dose of the Ad-sig-hMUC-1/ecdCD40L vaccine.<br>2) To determine the safety and efficacy of 5 injections of the vaccine given on Days 1, 8, 22, 52 and 82.
Eligibility	Carcinoma of the breast, colon, lung, ovary or prostate that has relapsed after initial regional therapy and has failed front line salvage therapy.<br><br>PS 0,1,2<br>Estimated survival > 12 months
Exclusion Criteria	History of Angina, IHD, AMI, stroke, TIA<br>Infectious Diseases<br>Carcinomatous meningitis

3. OBJECTIVES AND ENDPOINTS

3.1 Primary Objective:

- Characterize the safety profile of the Ad-sig-hMUC-1/ecdCD40L vector vaccine.
- Identify a tolerable, immunologically active dose level.
-

3.2 Secondary Objective:

- Evaluate patients' immune function pre- and post- treatment.

3.3 Exploratory Objectives:

- To evaluate the overall response rates (ORR) as assessed by RECIST v1.1

- To determine the efficacy of Ad-sig-hMUC-1/ecdCD40L vector vaccine as assessed by progression free survival (PFS)
- To determine the efficacy of Ad-sig-hMUC-1/ecd/CD40L vector vaccine as assessed by overall survival (OS)

4. BACKGROUND AND RATIONALE

4.1 MUC-1 in Cancers of the Breast, Colon, Ovary, Prostate and Lung

MUC-1 is a structural protein expressed at low levels on the apical surface of normal epithelial cells which functions to stabilize the protective layer of mucous. This protein marker has been shown to be highly overexpressed on neoplastic cells in 90% of cancers of the breast, ovary, colon and lung. [7-10] In epithelial cancer, MUC-1 overexpression is thought to disrupt E-cadherin function, leading to anchorage-independent tumor cell growth and metastases.[9-10] In animal models, suppressing the expression of MUC-1 reduces the rates of growth and metastasis and increases the sensitivity of the cancer to chemotherapy-induced cell death. Furthermore, overexpression of MUC-1 is independently correlated with adverse clinical phenotypes, metastases and resistance to chemotherapy.[8-17]

In cancer cells, the level of MUC-1 on the plasma membrane as measured by immunohistochemistry is increased in carcinomas of the breast, prostate, colon, ovary and lung.[18-25] Aberrant localization of the MUC-1 staining in the cytoplasm, rather than on the plasma membrane, has been shown to occur in 93% of invasive ductal carcinomas of the breast[7-14, 19-25] and to be correlated with worse prognosis. Circumferential staining in breast cancer cells, rather than on the apical membrane surface, has been correlated with an increase incidence of lymph node involvement.[18-19] Patients whose tumors show MUC-1 staining in more than 75% of the tumor cells have a decreased survival.[20]

Ninety-four percent of prostate cancers have been shown to be positive for MUC-1 expression by immunohistochemistry.[21] The diffuse pattern of expression of MUC-1 (uniform throughout the tumor) occurs in 62% of prostate cancer overall, in 9% of Gleason Grade 2, 64% of Gleason Grade 3, 80% of Gleason Grade 4, and 100% of Gleason Grade 5.[21] Gene expression profiling studies has shown that elevated MUC1 expression was a strong predictor of tumor recurrence independent of tumor grade.[22]

Analysis of diagnostic biopsy specimens from 93 cases of non-small cell carcinoma of the lung have shown that depolarized expression of MUC-1 is correlated with poor prognosis among the lymph node positive patients.[23] Immunohistochemical analysis of the biopsy specimens from 113 cases of carcinoma of the colon showed that the levels of MUC-1 were significantly higher in carcinoma tissues than in normal colonic mucosa. The levels of staining positivity for MUC-1 in tumor biopsies of patients with metastatic tumors were higher than in patients without metastatic disease.[24]

Elevated levels of MUC-1 expression by immunohistochemistry was found in 92%.[24-25] of the biopsy specimens from patients with epithelial ovarian cancers.[25] A significant association was found between high levels of MUC-1 expression by immunohistochemistry with histological grade and disease stage.[25]

The level of MUC-1 has also been found to be elevated in the clonogenic cells of cancer populations, often referred to as the cancer stem cells.[26-28] These cells exhibit chemotherapy resistance associated with pro-survival signals and increased proliferation. Thus, cancer cells which over-express the MUC-1 antigen appear to be the cancer cells which are contributing to relapse. Importantly, MUC-1 has been found to be on the TIC.

Interestingly, MUC-1 is highly immunogenic in mouse models and human clinical models (see work from Deisseroth laboratory).[29-36] Moreover, since the pattern of glycosylation is changed on the cancer cell, MUC-1 provides a cancer-specific antigen for immunization. This trial therefore

seeks to evaluate the safety and optimal dosing for a novel adenoviral vector vaccine that targets the hypoglycosylated MUC-1 antigen.

MUC-1 has been studied clinically as the target of vaccines by previous researchers.[11-17] MUC-1 has been packaged in liposomes, delivered as adenoviral vectors and given directly along with adjuvants. To date, there have been no toxic events associated with the administration of MUC-1 as a vaccine. Importantly, no previous group has linked MUC-1 to a strong activating signal, such as the CD40L The adenoviral vector used here works by programming the cells it infects to release the hMUC-1/ecdCD40L protein over a 10-14 day period.[29-36] The hMUC-1/ecdCD40L induces an immune response against hMUC-1 by binding to the CD40 receptor on dendritic cells (DCs). The binding of the CD40L to the CD40 receptor activates the DCs and results in the internalization of the hMUC-1/ecdCD40L protein which leads to presentation of the hMUC-1 antigens on Class I MHC. This leads to activation and maturation of antigen naïve CD8 cells to hMUC-1 specific CD8 effector cells in young individuals but this response is lost or diminished in the fifth and sixth decades of life.

4.2. Clinical Experience with CD40L Vaccines and Antibodies to the CD40 Receptor

Previous clinical studies have shown no serious adverse events associated with the subcutaneous injection of fibroblasts infected with an adenoviral vector carrying the CD40L transcription unit.[37] Other clinical studies have shown that the intravenous injection of CLL cells infected with the an adenoviral vector carrying a CD40L transcription unit was not associated with serious side effects.[38] These clinical studies suggest that the subcutaneous injection of the hMUC-1/ecdCD40L protein vaccine or the Ad-sig-hMUC-1/ecdCD40L vector vaccines may not cause significant side effects.

As shown below in Table I, three different agonistic monoclonal antibodies to the CD40 receptor have been introduced into Phase I and Phase II clinical testing for the treatment of cancer without major toxicity.[39-40] In addition, the CD40L itself has been tested clinically at the phase I level without major side effects.[41] Thus, the clinical testing of the components of the TAA/ecdCD40L have been shown by clinical testing to be safe, and extensive clinical studies about the activation of the CD40 receptor have been found to be safe and effective in the cancer vaccine area.

Table 1: Report of Clinical Trials of CD40 Antibody, CD40L and CD40L Producing Cells

Completed Trials for CD40 Agonistic Antibody Injected Intravenously:

Type of Trial	Drug Name	Company	# Patients Treated	# Trials
Phase I	SGN40	Seattle Genetics	132 treated	4
Phase I	HCD122	Novartis	68 treated	2
Phase I/II	SGN40	Seattle Genetics	12 treated	1
Phase II	SGN40	Seattle Genetics	32 treated	1
Total Patients Treated So Far			244	8

Trials Underway for CD40 Agonistic Antibody Injected Intravenously:

Type of Trial	Drug Name	Company	# Patients Projected	# Trials
Phase I	SGN40	Seattle Genetics	105 Projected	3
Phase I	CD870893	Pfizer	21 Projected	1
Phase I	Alvocidib	NCI	58 Projected	1
Phase II	SGN40	Seattle Genetics	240 Projected	1
Total Projected			424 Projected	6

Trials Completed for CD40L Injected Subcutaneously:

Type of Trial	Drug Name	Company	# Patients Treated	# Trials
Phase I	Avrend	Immunex	32 treated	1
Phase II	Recomb CD40L	NCI	5 treated	1

**Trials Completed for AdCD40L Transformed Cells:**		# Patients Treated	# Trials
Phase I	AdCD40L Infected CLL Cell IV	11 treated	1
Phase I	CD40L/IL2 Transformed Fib SC	10 treated	1

4.3. Description of the Ad-sig-ecd-hMUC-1/ecdCD40L Vaccine

In order to administer our vaccine in a way that could affect T cells in secondary lymphoid tissue

all over the body, and to create a therapy which does not require cytokine boosts (such as IL-2 conjugated antibody), we constructed an adenoviral vector encoding a chimeric tumor associated antigen (TAA) bound to an immune stimulant[29-36] In this vaccine, the TAA is the human MUC-1 epithelial antigen (hMUC-1), and the immune stimulant is both the adenoviral vector itself as well as CD40 ligand (CD40L). The adenoviral vector's transcription unit encodes a direct tandem repeat (each of which is 20 amino acids long) taken from the extracellular domain (ecd) portion of the hMUC-1 self antigen attached to the 209 amino acid ecd of the CD40L through a 14 amino acid linker. The ecd region of CD40L contains all of the sequences necessary for the formation of the CD40L trimer.[42]This transcription unit is preceded by a secretory sequence (sig) so that the cells infected by the Ad-sig-ecd-hMUC-1/ecdCD40L vector at the injection site will release the hMUC-1/ecdCD40L protein over 10 to 14 days. Research has shown that the use of the Ad-sig-hMUC-1/ecdCD40L adenoviral vector (rather than the hMUC-1/ecdCD40L protein) is the most potent initial stimulus for activation of the immune response to the hMUC-1.[29-36]

CD40L is one of the strongest "adjuvants" to the induction of an immune response that is known. CD40L is transiently expressed on activated CD4+ T-cells and B cells. Its presence is absolutely necessary for the upregulation of chemokine receptors like CCR7 in professional antigen presenting cells known as dendritic cells (DCs) leading them to mature and migrate to the regional lymph nodes, and to upregulate co-stimulatory signals (intercellular adhesion molecules which lead to sustained expansion of T cells). The binding of the chimeric hMUC-1/ecdCD40L to the CD40 receptor on the DCs leads to internalization of the hMUC-1. Internalized hMUC-1 protein is then processed on the Class I major histocompatibility complex (MHC) pathway and ultimately presented on the DC's Class I MHC. When the hMUC-1 protein is expressed on the surface of DCs in association with Class I MHC, CD8 effector cells are able to recognize the antigen and are stimulated to expand. In a similar fashion, CD40L leads to costimulation of macrophages. The expression of the CD40L is necessary for development of a humoral immune response as well.

Using CD40L in the vaccine might be particularly useful in older patients. Evidence generated in over 60,000 recipients of influenza A vaccine has shown that the neutralizing antibody response in vaccinated individuals over the age of 55 is less than 20% while that below 55 years is 80%.[43-44] This low level of response among older individuals is thought to be due to the low level of CD40L on activated CD4 cells from older individuals.[45] Thus, providing the CD40L in the vaccine circumvents this functional defect present among older individuals.

We have found that two subcutaneous (sc) Ad-sig-hMUC-1/ecdCD40L vector injections delivered at 7 day intervals can induce immunity which suppresses the growth of hMUC-1 tumor cells in 100% of the vaccinated mice[29, 31] without IL2 stimulation being required. A third vaccination with the Ad-sg-MUC-1/ecdCD40L vector further stimulated the immune response over that achievable with two vector injections.[31] However, our studies showed that the use of two sc injections of the hMUC-1/ecdCD40L protein (14 days apart) 7 days following the sc injection of the Ad-sig-hMUC-1/ecdCD40L vector injection (i.e., vector injections on Day 1, 8 and 22) produced higher levels of hMUC-1 specific antibodies and CD8+ effector cells than was the case with Ad-sig-hMUC-1/ecdCD40L vector injections alone (see Figures 2-3 below).[31] This method is called the Vector Prime Protein (VPP) regimen. Furthermore, three sc injections of the vector or the vector followed by two protein boosts completely prevent the engraftment of hMUC-1 positive mouse lung cancer cell line[31].

Pre-clinical results from the laboratory of Dr Albert Deisseroth (discussed below)[29-31, 34] have shown that subcutaneous injection of an adenoviral vector carrying a tumor associated antigen (TAA)/ecdCD40L fusion gene generates immunological resistance to TAA positive cancer cells for at least one year.

4.4. Relevant Pre-Clinical Studies

We have tested out the Ad-sig-TAA/ecdCD40L vector prime/TAA/ecdCD40L protein boost vaccine strategy with three different target associated antigens (TAA): the hMUC-1, the rat Her-2-Neu (rH2N), both of which are antigens overexpressed in breast cancer tissue, and one oncogenic viral associated antigens: the human papilloma virus E7 (which is the cause of

cervical cancer). Below is a review of the preclinical data generated in the two models relevant to this clinical trial.

4.4.a. Studies with the Ad-sig-hMUC-1/ecdCD40L Vector Prime-hMUC-1/ecdCD40L Protein Boost Vaccine Strategy in hMUC-1.Tg Mice.

One model we used to test the vector prime-protein boost vaccine strategy is the hMUC-1.Transgenic (hMUC-1.Tg) mouse model. These mice carry a gene encoding the human MUC-1 gene from birth and therefore are tolerant (immunologically unresponsive) to the hMUC-1 protein and thus to the hMUC-1 positive mouse cancer cells[36]. These transgenic mice were injected subcutaneously with adenoviral vector carrying the ecdhMUC-1/ecdCD40L fusion gene. Seven days later, the mice were then injected with the hMUC-1 positive tumor cell line LL2/LL1hMUC-1. As shown in Figure 1, the hMUC-1 positive cancer cell line did not grow in the vaccinated mice.[29-31] The induction of in vivo resistance to the growth of the hMUC-1 positive syngeneic mouse cancer cells was shown to involve a CD8+ T cell lymphocyte immune response against the hMUC-1 self antigen.[31] Thus, the hMUC-1/CD40L vector injections appeared to overcome immune tolerance.

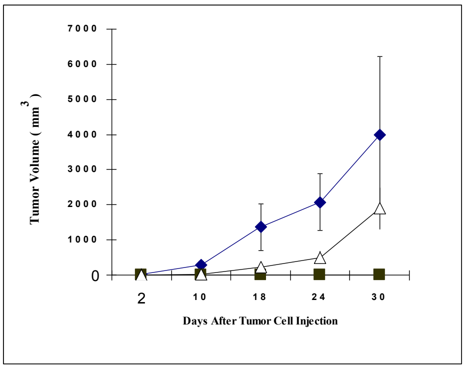

Figure 1: Ad-sig-hMUC-1/ecdCD40L Vector Vaccine Which Encodes Epitope for Subunit I (All Extracellular) of the hMUC-1 Linked to the ecd of the CD40L Suppresses Growth of the LL2/LL1hMUC-1 Cell Line in hMUC-1.Tg Mice. Test mice were injected sc with the Ad-sig-hMUC-1/ecdCD40L vector prime and hMUC-1/ecdCD40L protein boost vaccine and then injected sc with the LL2/LL1hMUC-1 tumor cells ("Prevention" Experiment). We then measured the size of the sc nodule which developed at the sc injection site of following sc injection of 500,000 LL2/LL1hMUC-1 Tumor Cells in hMUC-1.Tg mice which had been vaccinated with the Ad-sig-hMUC-1/ecdCD40L vector. The following was used for the vaccination: No vaccination (solid diamonds); Ad-sig-hMUC-1/ecdCD40L Subunit I vector (solid squares); Ad-sig-hMUC-1 Subunit I vector (open triangles).

4.4.b. Effect of hMUC-1/ecdCD40L Protein Boost on Murine Immune Response.

In order to test the effect of boosting the vector vaccination with the subcutaneous protein injections, we studied the effect of various schedules of subcutaneous injections of the Ad-sig-hMUC-1/ecdCD40L vector followed by 2 sc injections of the hMUC-1/ecdCD40L protein on the induction of a hMUC-1 specific humoral and cellular immune response, and on the growth of the LL1/LL2hMUC-1 tumor cell line in a hMUC-1 transgenic mouse model. When two subcutaneous injections of the hMUC-1/ecdCD40L protein, 14 days apart, were given 7 days following the sc injection (on Days 1, 8 and 22) of the Ad-sig-hMUC-1/ecdCD40L vector (the VPP or T5 regimens), the level of immune response (as measured by the levels before and after vaccination of hMUC-1 specific T cells or hMUC-1 specific antibodies) against the hMUC-1 antigen in the hMUC-1.Tg mice was enhanced dramatically (see T5 in Figures 2 and 3).

These data showed that the VPP or T5 regimens induced an immune response to the hMUC-1 antigen in a transgenic mouse strain which is normally tolerant to that antigen. In conclusion, hMUC-1.Tg mice were able to suppress the growth of hMUC-1 positive tumor cells after receiving hMUC-1 vaccine and protein boosters. These data suggest that an Ad-sig-hMUC-1/ecdCD40L vector prime-hMUC-1/ecdCD40L protein boost vaccine might be useful in the 80% of breast cancers that overexpress hMUC-1.

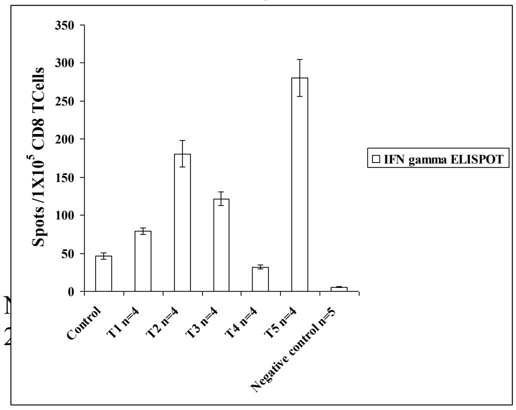

Figure 2: The Effect of Various Schedules of the Ad-sig-hMUC-1/ecdCD40L Vector and the hMUC-1/ecdCD40L Protein on the Level of Interferon-Gamma Positive T Cells in the Spleen of hMUC-1.Tg Mice Before and After Vaccination. The following combinations of the Ad-sig-hMUC-1/ecdCD40L vector (V) and

hMUC-1/ecdCD40L protein (P) were injected sc into the hMUC-1.Tg mice: Each sc administration was conducted at 7 day intervals. Control=VVNN; T1=VVPN; T2=VVNP; T3=VPNN; T4=VNPN; T5=VPNP; Negative Control=NNNN. N=nothing. All injections (A, P, or N) are separated at 7 day intervals. V=Vector; P=Protein; N= Nothing.

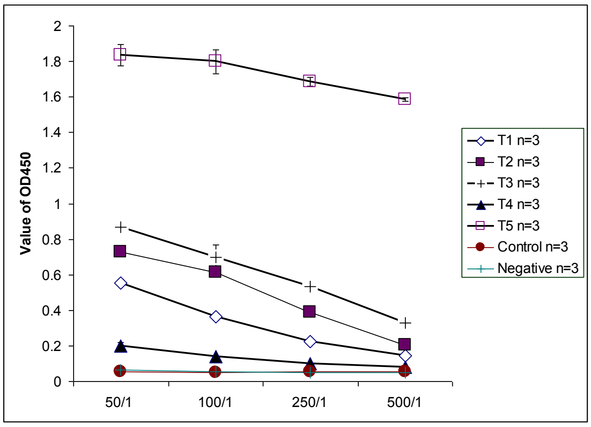

Figure 3: The Effect of Various Schedules of the SC Injection of the Ad-sig-hMUC-1/ecdCD40L Vector and the hMUC-1/ecdCD40L Protein on the Level of hMUC-1 Specific Antibodies in ecdhMUC-1.Tg Mice. The following combinations of the Ad-sig-hMUC-1/ecdCD40L vector (V) and hMUC-1/ecdCD40L protein (P) were injected sc in the hMUC-1.Tg mice: Control=VVNN (solid circles); T1=VVPN (open diamonds); T2=VVNP (solid squares); T3=VPNN (bold line+bold line); T4=VNPN (solid triangles); T5=VPNP (open squares); Negative Control=NNNN (thin line+thin line). N=nothing. All injections are at 7 day intervals. V=Ad-sig-hMUC-1/ecd CD40L vector; P=hMUC-1/ecdCD40L protein

4.4.c. Binding of the hMUC-1 Antibodies Induced by Vaccine in hMUC-1.Tg Mice to Biopsy Samples of Human Breast Cancer.

We then tested if the hMUC-1 antibodies induced in the hMUC-1.Tg mice by the Ad-sig-hMUC-1/ecdCD40L vector prime and hMUC-1/ecdCD40L protein boost (Subunit I) VPP vaccination would bind to human breast cancer epithelial cells. The antibodies from the Ad-sig-hMUC-1/ecdCD40L vaccinated mice bound to 54 of the 100 of the breast cancer specimens tested (see examples in Panel I of Figure 4). In addition, exposure of the mouse serum to the specific hMUC-1 20 AA repeat peptide encoded by the vector or protein transcription units blocked completely the binding of the mouse antibodies to the breast cancer cells (see Panel II of Figure 4). Serum from unvaccinated mice did not bind to the human breast cancer cells (see Panel III of Figure 4). The AA sequence of the hMUC-1 peptide was then scrambled so that the order of the AA was randomized but the composition of AA remained the same. This peptide did not block the binding of the serum from the vaccinated hMUC-1.Tg mice (data not shown).

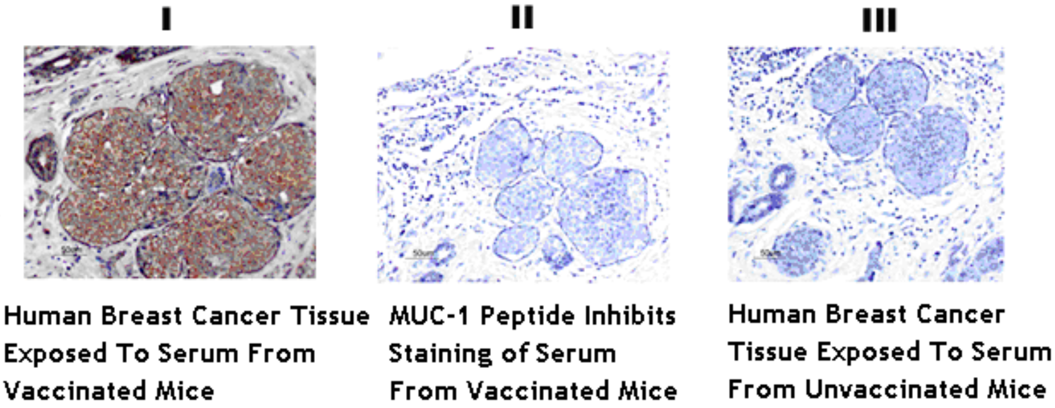

Figure 4: Binding of Antibodies from the Serum of Ad-sig-hMUC-1/ecdCD40L Subunit I Vector Vaccinated Mice to Human Breast Cancer Cells. Serum collected from hMUC-1.Tg mice following vaccination with the Ad-sig-hMUC-1/ecdCD40L vector and hMUC-1/ecdCD40L protein was applied to sections from human breast cancer clinical specimens. Panel I: Antibodies from vaccinated mice. Panel II: Antibodies from vaccinated mice which were exposed to the hMUC-1 specific peptide used in the vaccination prior to applying the mouse serum to the sections; Panel III: Serum from unvaccinated mice.

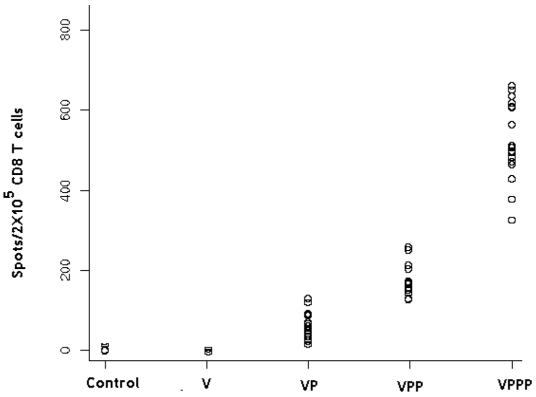

Figure 5

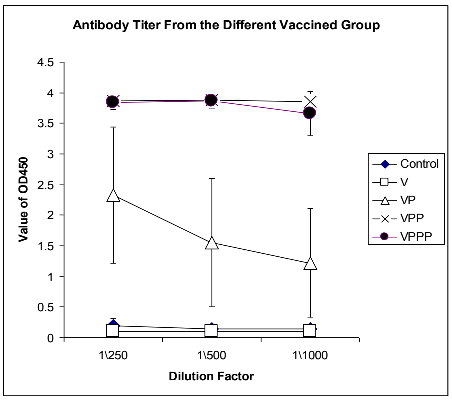

Figure 6

Figures 5 and 6: Effect of Varying Number of Protein Boosts on Level of E7 Specific CD8 T Cells in the Spleens of Vaccinated C57Bl/6 Mice. Mice were injected sc with nothing (control) or one Ad-sig-E7/ecdCD40L vector, or that vector plus one, two or three sc E7/ecdCD40L protein boost injections, at 7 day intervals between the injections. 7 days later, the mice were sacrificed, and the spleen cells analyzed for E7 specific CD8 T cells by ELISPOT assay (Figure 5) and the serum was analyzed for the level of E7 specific antibodies by ELISA assay (Figure 6).

4.4.d. Use of the Ad-sig-E7/ecdCD40L Vector Prime-E7/ecdCD40L Protein Boost Vaccination in Old (18 Months) vs Young (2 months) Old Mice.

The second relevant antigen tested was the E7 antigen of the human papilloma virus. This is a foreign antigen, in contrast to the hMUC-1, which is a self antigen. We used the Ad-sig-E7/ecdCD40L vector prime-E7/ecdCD40L protein boost to test two questions: how many boosts are necessary to induce an increase in antibodies and CD8 T cells specific to the target antigen (E7) and does the VPP vaccine work in older mice as well as younger mice. A viral antigen is easier to use than the hMUC-1 antigen which requires the breeding of transgenic mice. As shown in Figures 5-6 (see above), two or three protein boost injections (the vector and protein boosts being administered at 7 day intervals) dramatically increases the level of the E7 specific antibodies and the E7 specific CD8+ T cells

As outlined above, the immune response in breast cancer patients who are 55 years or greater, may be diminished[43-44] due to fewer numbers of both CD4+ and CD8+ antigen- naïve T cells, and also due to the presence of a functional defect in the CD4+ cells via underexpression of the CD40L. As all the experiments described above involved 2-month old mice, we used the Ad-sig-E7/ecdCD40L vector prime-E7/ecdCD40L protein boost to test the magnitude of the immune response induced in old (18 month) mice and compared this to young (2 month old) mice. Two month (young) or eighteen month (old) C57Bl/6 mice (equivalent in chronological age to human beings 55 years or greater in age) were vaccinated with the VPPP regimen (1 sc injection of the Ad-sig-E7/ecdCD40L vector followed by 3 sc injections of the E7/ecdCD40L protein).

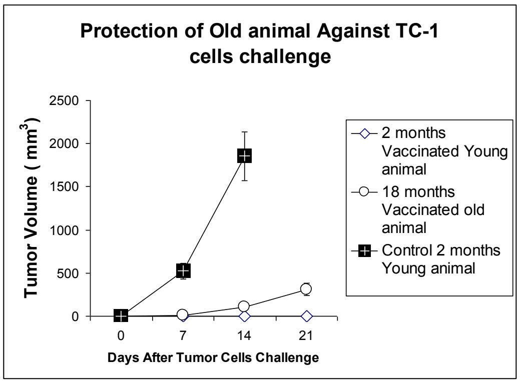

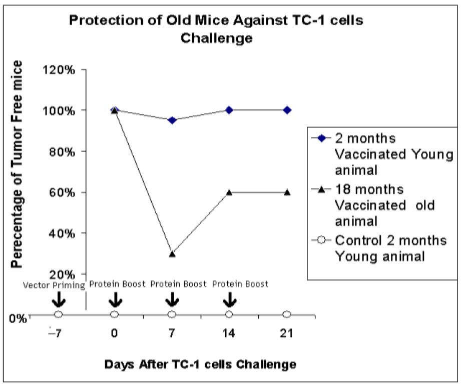

Figure 7 **Figure 8**

Figures 7 and 8. Effect of the Ad-sig-E7/ecdCD40L Vector Prime/ E7/ecdCD40L Protein (3X) Boost (VPPP) Vaccine Against the E7 Viral Antigen in Old (18 Months) Mice on Growth of E7 Positive TC-1 Tumor Cells. C57BL/6J mice were injected once sc with the Ad-sig-E7/ecdCD40L vector and then 3 times sc with E7/ecdCD40L protein injections (every 7 days) starting 7 days after the vector injection. Ten micrograms of the E7/ecdCD40L protein boost were used for each injection. The tests were carried out in 18 months old mice, or 2 months old mice. The results are expressed as the change in the volume of the subcutaneous nodules of the TC-1 cells (Figure 7) or the percentage of mice which are tumor free at any point following the tumor injection and vaccination (Figure 8).

As shown above in Figure 7, the suppression of the TC-1 tumor cell line growth in the old vaccinated mice is almost as good as in the young mice. Importantly, as shown in Figure 8, the use of E7/ecdCD40L protein boosts can suppress the growth of E7 positive tumor in "progressor mice" in which the tumor is established and growing.

4.4.e. Discussion of Results of Preclinical Data.

The results of the experiments reported above show that the injection of the Ad-sig-E7/ecdCD40L vector into C57BL/6 mice induces immune resistance to the engraftment and growth of E7 positive tumor cells. The Ad-sig-E7/ecdCD40L also induces regression of established subcutaneous E7 positive tumor nodules in the C57BL/6 mice. Intraperitoneal injection of splenic CD8 T cells collected from Ad-sig-E7/ecdCD40L vaccinated mice which had remained tumor free for over one year following injection of the Ad-sig-E7/ecdCD40L vector and tumor challenge, induced regressions of TC-1 tumors already growing in immunocompromised athymic nude recipient mice. This experiment shows that the effect of the Ad-sig-E7/ecdCD40L injections on E7 positive TC-1 cells is mediated by a CD8+ T cell dependent immune response which lasts for over one year.[29]

We also evaluated whether vaccination of hMUC-1.Tg mice with the Ad-sig-ecdhMUC-1/ecdCD40L vector alone could activate a CD8+ T cell lymphocyte immune response against the hMUC-1 positive tumor cells. This proved to be the case as shown by the data presented in Figures 1-3. To summarize, the Ad-sig-TAA/ecdCD40L vector strategy is unique in several ways. It has been shown to break tolerance in a transgenic mouse model without the use of cytokine boosting. In addition, it can generate cellular immunity for up to a year, which indicates that the vector strategy outlined in this paper induces memory cells. Importantly, the subcutaneous injections of the ecdhMUC-1/ecdCD40L protein boost can be used to dramatically enhance the induction of the immune response in the anergic animals, even when they are old. This suggests that the Ad-sig-hMUC-1/ecdCD40L vector prime-hMUC-1/ecdCD40L protein boost has the potential to be an effective vaccine in human breast cancer. As outlined above, the MUC-1 has been tried in the past which only modest success. MUC-1 is immunogenic but the immune response was not as great as in the current studies in which the hMUC-1 was linked to the CD40L and then implanted in the adenoviral vector.

5. STUDY DESIGN

5.1 Type of Study.

This is a single-site phase I non-randomized open label dose escalation trial of the Ad-sig-hMUC-1/ecdCD40L vector vaccine (MUC-1 vector vaccine) for men or women with metastatic or recurrent breast, prostate, colon, ovary or lung, who have been on their current therapy or on no therapy for a one month period prior to enrolling in this trial, and who have had an elevated serum MUC-1 level (CA15-3) at some point since their initial diagnosis of epithelial cancer. This vaccine is directed to MUC-1, which is a marker for poor prognosis.[16-25]

Part 1 of this trial is a 3+3 dose escalation phase 1 toxicity trial in which patients will receive a single subcutaneous administration of the Ad-sig-hMUC-1/ecdCD40L vector vaccine in cohorts 1-4. The primary objective of part 1 was to determine the MTD of the Ad-sig-hMUC-1/ecdCD40L vector when administered once to each participating subject. Subsequently in Part 2 which contains cohorts 5 and 6, the primary objective is to test for toxicity and efficacy of two successive administrations of the vaccine (seven days apart) in Cohort 5 and three successive administrations of the vector 7 and 14 days apart in cohort 6 (on Days 1, 8 and 22) (see Table 2). In Part 3, six monthly injections will follow an initial 3 subcutaneous vector vaccine injections at Days 1, 8 and 22 in order to determine the number of monthly injections of the vaccine which are necessary for the regression of tumor nodules.

Subsequent to signature of informed consent, eligible subjects with measurable or non-measurable metastatic epithelial cancer will receive a single dose of subcutaneous MUC-1 vector vaccine in cohort 1-4, two subcutaneous injections of the MUC-1 vaccine (7 days apart) in dose cohort 5, and three subcutaneous injections of the MUC-1 vaccine on days 1, 8 and 22 in cohort 6. In cohorts 5 and 6, the dose of the vaccine will be the highest tolerable dose among the initial 4 cohorts. The time expected to enroll and treat a minimum of 6 and maximum of 24 patients in cohort 1-4 is approximately 56 weeks whereas approximately 52 weeks is expected to enroll and treat a minimum of 3 and maximum of 24 patients in cohort 5-6. Six patients will be treated in Cohort 7 with vector injections on Days 1, 8, 22, 52 and 82.

The emphasis of cohorts 1-6 of this trial is safety and tolerability. Serum MUC-1 levels and changes in the function of the immune system before and after vaccination will also be evaluated. The goal of Cohort 7 is to determine the safety and efficacy of 5 vector injections.

.

The purpose of the clinical trial is to test the safety of a single dose of the Ad-sig-ecdhMUC-1/ecdCD40L vector vaccine in cohort 1-4, two doses of the vector vaccine given 7 days apart in cohort 5, three doses of the vector vaccine given 7 and 14 days apart on Days 1, 8 and 22 in cohort 6 and then in Part 3, 5 vaccinations will be given on Days 1, 8, 22, 52 and 82. Secondarily the efficacy of the Ad-sig-MUC-1/ecdCD40L vector will be evaluated by monitoring changes in the serum MUC-1 levels (using CA15-3 assays for determination of serum levels of MUC-1 shed from the tumor cells) and immunological analysis of the peripheral blood before and after vaccination. Although not useful for diagnosis due to limitations of sensitivity and specificity, the CA15-3 is useful clinically to follow the response to therapy. Finally, CT and PET scans will be used to detect regressions of tumor nodules. Subjects will be continued to be followed until disease progression, intolerable toxicity, elective withdrawal from the study or study termination. Upon disease progression, subjects will be followed every 3 months for survival and details of post-progression therapy.

5.2 Treatment Plan.

This trial has six initial cohorts with three subjects planned for each of these initial cohorts, and 6

patients in Cohort 7. Prior to the start of treatment, all subjects will be evaluated for eligibility and must have a signed informed consent. The patients will be entered sequentially in Cohorts 1-7. There will be within each cohort a 2 week interval between day on which a patient received the last vaccination and the day on which the next patient within that cohort receives the first vaccination for Cohorts 1-6. There will be 4 week between the last vaccination of the last patient entered on a cohort and the first patient entered on the next higher cohort.

Subjects in the first cohort will receive 1 dose of vaccine injection at the lowest planned dose of the vector, 1 x 10^9 VP. If none of the patients in the first cohort experiences dose limiting toxicity (DLT), a second cohort will receive 1 dose of 1x10^{10} VP. If none of the patients in the second cohort experiences DLT, the dose escalation will continue with the third cohort receiving 1 dose of 5 x 10^{10} VP per injection on day 1. The patients in the fourth cohort will receive 1 injection of 1x10^{11} if no DLT occurs in the preceding cohort. If no DLT occurs in the three patients enrolled in Cohort 4, then that will be the dose used in the next two cohorts.

Three patients will be enrolled in cohort 5 in which two subcutaneous injections 7 days apart of the highest dose (e.g. 1x10^{11} viral particles (VP) 7 days apart) of the vector which was tolerated in the 4 cohorts will be given to each patient (Days 1 and 8). If no DLT occurs in the first three patients enrolled in cohort 5, three additional patients will be enrolled in cohort 6 in which three injections of the vector (e.g. 1x10^{11} VP/injection) will be given to each of 3 patients on Days 1, 8 and 22. If no DLT occurs in the first three patients enrolled in cohort 6, then 6 patients will be entered into Cohort 7 in which each patient will receive 5 vector injections on Days 1, 8, 22, 52 and 82.

DLT will be defined as grade III or greater toxicity not definitely related to the disease process (or grade II or greater allergic or autoimmune reactions not definitely related to the disease process) within the first 4 weeks post vaccination.

Dose escalation decision will be made after all patients in each cohort have been observed for at least 4 weeks post vaccination. Any grade IV toxicity will prompt consideration for trial termination.

In Cohorts 1-4 if 1 patient out of 3 experienced DLT, the cohort will be expanded to 6 patients. If 2 or more (if the cause is determined to be the vector vaccine) is observed in these 6 patients, the next lower dose cohort will be expanded to 6 patients; if only </= 1 patient out of the 6 had DLT, this lower dose will be the Maximum Tolerated Dose (MTD). If no more than a total of 1 DLT (out of six subjects) is observed in this cohort, the study will proceed onto the next dose level. If two or more patients show DLT at any dose level, further injections of all other patients will be done at the previous dose level.

If two or more DLTs are observed in the first dose cohort, the dose level will be 50% of the initial starting dose. In the event we observe 2 / 6 DLT in the second dose level (1x 10^{10} VP) and 0 / 6 DLT in the first dose level (1x 10^9 VP), the study protocol may be submitted to CIRB for amendment to include an intermediate dose level (i.e., 5x 10^9 VP).

If 1 patient out of the first 3 patients of cohorts 5-6 experiences a DLT, the cohort will be expanded to 6 patients. If no more than one DLT occurs in the six patients in that cohort (cohort 5), then the trial will proceed to the next higher cohort (cohort 6). If no more than 1 patient out of six (or zero out of 3 patients) experience a DLT in cohort 6, then the dose and schedule of injections in that cohort will be the dose used in further trials (e.g. phase 2) of this vaccine.

If 2 or more DLT (if the cause is determined to be the vector vaccine) is observed in these 6 patients in cohort 5, two injections 7 days apart at the dose level which is 2 fold lower than the starting dose will be tested for each of two injections 7 days apart in each of 3 patients. If no DLT are observed, then, testing will proceed to cohort 6.

If 2 or more DLT (if the cause is determined to be the vector vaccine) is observed in this second set of 6 patients in cohort 5 using the 2 fold dose reduction, then a four fold dose reduction of the two injections 7 days apart at the dose level will be tested in 3 patients. If no DLT are observed,

then entry of patients into Cohort 7 will begin. If 2 or more DLT (if the cause is determined to be the vector vaccine) is observed in this third set of 6 patients in cohort 5 using the 4 fold dose reduction, then the dose and schedule of cohort 4 will be the dose and schedule for the next trial.

If 2 or more DLT is observed in these 6 patients in cohort 6, then a 2 fold reduction will be implemented for each of 3 injections given at Days 1, 8 and 22. If no or 1 DLT is seen in 6 patients, then that will be the dose and schedule for future trials.

If 2 or more DLT (if the cause is determined to be the vector vaccine) is observed in these 6 patients in cohort 6, then a 4 fold reduction of the dose will be implemented for each of 2 injections 7 days apart followed by a third injection 14 days later at the dose level which is 4 fold lower than the starting dose will be tested for the injections given on Days 1, 8 and 22 in each of 3 patients. If no DLT are observed, then that will be the dose and schedule for the next trial.

If 2 or more DLT (if the cause is determined to be the vector vaccine) is observed in this second set of 6 patients in cohort 6 using the 4 fold dose reduction, then the dose and schedule followed in cohort 5 will be the dose and schedule for future trials.

If any patient within Cohort 7 experiences a Grade 3 or higher adverse event (causality is assessed to be study treatment related), then that patient will not receive any additional vaccinations. If any patient in Cohort 7 experiences Grade 2 or greater allergic or autoimmune reactions not definitely related to the disease process within 4 weeks of any vaccination, then vaccinations in that patient will be discontinued.

If any subject in cohorts 1-7 experiences grade 1 or grade 2 adverse event which is non-study treatment related after each vector injection, the subsequent dose may be delayed up to 7 days.

If any subject in cohorts 1-7 experiences grade 1 or grade 2 adverse event (causality is assessed to be study treatment related) after each vector injection, the subsequent dose may be given if the adverse event resolves within 7 days.

If the adverse event does not resolve within 7 days, the subsequent injection may be discontinued. Exception can be applied on a case by case basis after discussion between principal investigator and MicroVAX.

In the event that there is dose delay, the subsequent injection will be delayed accordingly based on the initial injection interval. Subsequent scheduled follow-up visits will be delayed accordingly with respect to the last vaccination too.

The entire study may be discontinued by the study investigators, SCRI, NCCS, MicroVAX or the FDA. Study investigators and MicroVAX reserve the right to terminate the study (or re-design the protocol) at any time for any reason. Criteria for study discontinuation due to toxicity may include:

- Grade III toxicity attributable to the study agent in more than 3 patients.
- Grade IV toxicity or death attributable to the study agent(s) in any subject at any dose level.

5.3 Ad-sig-hMUC-1/ecdCD40L Vector Administration

Eligible subjects who have signed an informed consent will receive the vector as subcutaneous injection over the anterior thigh, shoulder or forearm of the patient. For details of the administration, please see Section 9.3.

5.4 Primary Objectives

Characterize the safety profile of the Ad-sig-hMUC-1/ecdCD40L vector vaccine. Identify a tolerable, immunologically active dose level. Finally, in Cohort 7, the goal is to determine the safety and efficacy of 5 vector injections given at a dose of 1x10E11/dose.

5.5 Secondary Objectives: Determination of the Optimal Biological Dose

Since preclinical data suggests that we may not encounter toxicity and therefore may not be able to define a MTD, we are carrying out a number of mechanistic assays to determine the optimal biological dose of the vaccine. The optimal biological dose is the dose that is associated with induction of an immune response as defined by in vitro cellular assays (using mononuclear cells from participants' blood as well as antibody assays on the serum of vaccinated patients).

Subjects may not have measurable disease by RECIST criteria (eg good partial response or complete response following therapy) and thus may not be evaluable for clinical response by imaging. However, if a patient's serum MUC-1 (i.e., CA15-3) levels decrease by more than 50 units and remain at that level or lower for four weeks after the vaccination, it will be concluded that the vaccine has *potential* efficacy.

Elevations in CA15-3 serum levels are seen in 75% of patients with breast cancer. Normal serum levels for CA 15-3 range from 9-51 units/ml and elevated levels of CA 15-3 are found in 63% of patients with lung cancer and 80% of patients with ovarian cancer. Normal conditions that are associated with increased CA 15-3 levels include: ovarian cysts, endometriosis, first trimester pregnancy, benign breast disease, kidney disease and liver disease. Five percent or less among cancer free individuals will have an elevated CA 15-3. Over 90% of patients who have metastatic breast cancer have elevated CA 15-3 levels.

Patterns of response to immuno-oncology agents differ from those observed with drugs such as chemotherapy or molecularly targeted agents. There are limitations to conventional response assessments such as RECIST criteria which have been developed in the era of chemotherapy for which efficacy usually correlates with tumor shrinkage. Delayed but durable clinical responses associated with prolonged survival was observed in a subset of patients on immunotherapy. In addition, although the overall response rates in immune checkpoint inhibitor trials were modest; overall survival data with the use of new criteria of immune-responses was longer than historical median. The application of immune-related response criteria is therefore important to accurately capture efficacy of immuno-oncology agents. Following completion of vaccination, subjects will be followed for disease progression and survival through review of medical charts or imaging as obtained as per standard of care.

5.6 Expected number of subjects

Up to 54 patients will be enrolled into cohorts 1-7 of this trial.

6. STATISTICAL ANALYSIS

We are employing the traditional 3+3 dose escalation scheme in cohorts 1-6 of this Phase I clinical trial. Our primary endpoint is assessment of safety and toxicity, and the operating characteristics of this standard design relative to this assessment are well-established.[47-51]

6.1 Toxicity Evaluation

The dose escalation scheme in cohorts 1-6 is straightforward. Patients will be enrolled in cohorts 1-6 in groups of 3 to 6, in this sequential group, dose-escalating design. If none of the 3 patients in a given cohort experience DLT, dose escalation will proceed to the next level. If DLT occurs in one of the first three patients in a cohort, accrual to that cohort will continue up to a total of 6 patients or until 2 patients experience DLT, whichever comes first. If there are 0 or 1 DLT's experienced in this cohort, accrual to the next cohort will proceed.

The MTD is the maximum dose tested at which less than 2/6 experience DLT AND the dose above it experience >/= 2/6 DLT. Specific criteria for establishing the MTD are as follows:

DLT in 0 of 3 patients: proceed to next cohort
DLT in 1 of 3 patients: accrue up to 3 additional patients to this cohort
DLT in 1 of 6 patients: proceed to next cohort
DLT in 2 or more of 6: establish MTD at the dose of the previous cohort
DLT in at least 2 of 3 patients: establish MTD at the dose of the previous cohort

Dose limiting toxicities (DLTs) for this study will be defined as the following: "Any grade III or greater toxicity as defined by NCI v.4 CTC criteria which is not definitely related to the disease process. In addition, DLTs will include the occurrence of any Grade 2 or greater allergic reactions and Grade 2 or higher autoimmune reactions which is not definitely related to the disease process.

All patients in Cohort 7 will be treated with 5 administrations of the vector vaccine with the highest dose of the Ad-sig-hMUC-1/ecdCD40L vector which is tolerated in Cohorts 1-6. These vector injections will occur on Days 1, 8, 22, 52 and 82.

6.2 Response evaluation and assessment of disease status

Disease evaluation and imaging studies such as CT or MRI scan will be performed at baseline and at 2 months after last vaccination for cohorts 1-6 and on days 52, 112, 172 for cohort 7. Subsequently, disease evaluation will be performed as per institutional standard. Information on objective tumor assessments will continue to be collected through medical chart review until confirmed objective disease progression or start of new anticancer therapy whichever occurs first. The efficacy outcome for exploratory objectives are as follows:

ORR defined as subjects achieving complete response, partial response or stable disease as defined by RECIST 1.1

PFS as defined from date of study treatment start to date of disease progression, start of new anti-cancer therapy or death, whichever occurs first

7. PATIENT SELECTION

7.1. Inclusion Criteria

1. Men or women age 21 yrs or older with biopsy-proven recurrent or metastatic, measurable or non-measurable adenocarcinoma of the breast, ovary, lung, colon or prostate.
2. Elevated MUC-1 levels (as measured by immunohistochemistry on tumor biopsy or by serum tumor marker: CA 15-3 or CA27.29) at any time since the diagnosis of cancer (levels do not need to be elevated at the time of trial entry). .
3. Received at least one line of palliative chemotherapy
4. No chemotherapy and/or radiation therapy for at least 28 days before consent
5. Left ventricular ejection fraction greater than or equal to the lower limits of normal.
6. ECG with no evidence of ischemia or infarction.
7. Ability to understand the study's risks, benefits, and procedures and provide written informed consent.
8. Performance status $\leq$2 on the ECOG performance scale and life expectancy of greater than 12 months.
9. Acceptable pulmonary function. (If questionable a pulmonary function test will be performed.)
10. Negative serology for hepatitis B (HbSAg negative), hepatitis C and HIV.
11. All of the following: white blood count > 3500, absolute neutrophil count >/= 1,500, hemoglobin $\geq$ 8 g/dL, platelet count $\geq$ 100,000/dL: Bilirubin <1.5, AST, ALT < 2 times the upper limit of normal (AST, ALT < 3 times the upper limit of normal for subjects with liver metastases), and calculated creatinine clearance >/= 50 mls/min.

12. For women with child-bearing potential: negative urinary pregnancy test at screening and within 7 days of enrollment; for men or women: willingness to use an approved contraceptive method while participating in this trial. Documentation of type of contraception patient will be using must be included in screening visit note by investigator.

7. 2. Exclusion criteria

1. History of bronchospasm or asthma that requires steroid treatment (inhaled or oral).
2. Treatment with steroid (for any condition, except for chemotherapy premedication or emesis) within 28 days of trial registration.
3. Current anti-cancer treatment with doxorubicin (Adriamycin), lapatinib, trastuzumab, bevacizumab or other monoclonal antibody therapy. Patients must be off these therapies for at least 28 days. Patients may have been exposed to chemotherapy or radiation therapy 4 weeks prior to receiving the vaccination.
4. Current anti-cancer treatment with tamoxifen. Patients must be off tamoxifen for at least 28 days prior to enrollment. (Aromatase inhibitors and raloxifene are allowed)
5. History of any autoimmune disease such as lupus, rheumatoid arthritis or psoriasis.
6. Uncontrolled diabetes mellitus.
7. Unable or unwilling to undergo repeated clinical evaluations and other diagnostic procedures or unable to sign an informed consent.
8. History of other malignancies except squamous or basal cell carcinomas of the skin or cervical carcinoma *in situ*.
9. History of organ transplant or allogeneic bone marrow transplants.
10. Pregnant or nursing females.
11. Any acute or chronic viral, bacterial, or fungal infection which requires specific therapy. Acute therapy must be completed within 14 days prior to study treatment.
12. Any underlying conditions which would contraindicate therapy with study treatment (or allergies to reagents used in the study treatment).
13. Any history of cardiac disease including arrhythmia (requiring active treatment or medications), heart failure, angina, infarction or coronary artery disease.
14. History of hypercoagulable disorder including history of prior pulmonary embolism, antiphospholipid antibody syndrome, deep venous thrombosis (except for indwelling intravenous catheter associated DVT, as long as patient has completed anticoagulation therapy)
15. Any brain or leptomeningeal involvement by the cancer
16. Known Ornithine transcarbamylase deficiency

Note: Patients not meeting all inclusion and exclusion criteria may not be enrolled in this study. Should there be any question regarding patient eligibility, the final decision on study entry is to be made by the principle investigator (PI) of the trial and MicroVAX and written approval from the PI or MicroVAX must be obtained**.**

8. SCREENING EVALUATION,COUNSELING AND PATIENT CONSENT

8.1 Screening of patients

Their primary physicians in consideration for this study will refer patients. There is no intention to advertise this trial to the public. A screening log must be kept of all patients considered for the study and subsequently excluded; the reason for exclusion must be also be recorded. Screening procedures may be performed within 28 days after last chemotherapy.

8.2 Patient counseling

Patients who are referred for the Ad-sig-hMUC-1/ecdCD40L vaccine trial will have a counseling session with the clinical trial team to explain the rationale of the protocol and the potential risks in participating in this clinical trial. The cancer vaccination procedure as well as the side effects of the procedure will be explained to the patient. It will be pointed out specifically that cancer vaccination is still considered experimental but that there have been many trials with sc administration of adenoviral vectors with acceptable toxicity. There have also been many trials focused on the CD40L/CD40 receptor axis which have shown activity and acceptable safety. Patients will be informed that they retain the right to withdraw from the study at any time. The patient will be thoroughly evaluated with a detailed history and physical examination and a series of studies to confirm medical eligibility. Treatment recommendations and alternatives (as far as they exist) will then be discussed thoroughly with patient and family.

8.3. Cost Considerations

Cost of manufacturing and production of the cancer vaccine will be borne by MicroVAX, LLC. In addition, the costs of research bloods and analysis will also be borne by grants to National Cancer Centre Singapore. The cost of routine pre-vaccination blood investigations, follow up CT scan evaluations and x-rays will be borne by the patient. In the event that subjects are admitted to hospital from the course of their treatment, the cost of hospitalization will also be borne by the patient or his insurer.

8.4 Patient Consent

Patient Consent is obtained using institutionally-approved forms. Consent will be taken for pre and post-treatment tissue sample biopsy after a full discussion with the patient. In addition, consent for blood sampling for evaluation and research will also be discussed with the patient.

9. TREATMENT PLAN

9.1 Timing of enrollment

Patients who are at least 28 days post-chemotherapy and/or targeted therapy are eligible for this study.

9.2 Preparation of vaccine for injection in the clinic

The drug will be sent in one shipment: 18 vials of 5X10E9 VP/0.5mL and 48 vials of 5X10E10 VP/0.5mL from SAFC Pharma (GMP manufacturer) to the investigational pharmacy for first 6 cohorts.

Subsequently, the high dose vials ($5X10E^{10}$ VP/0.5mL in each vial) of the Ad-sig-hMUC-1/ecdCD40L vaccine (108 needed for Cohort 7) will be sent in one shipment from SAFC Pharma (GMP manufacturer) to the investigational pharmacy in Singapore. The dry ice filled box containing the vials will be covered in Mylar to protect the drug from the CO_2 released by the dry ice. Upon arrival the box containing the vials should immediately be placed in a limited access locked minus eighty freezer with a temperature monitor. The drug is to be kept at ≤ -60ºC at all times.

The vial number along with the date and time of administration will be recorded both on the patient record as well as in the hospital investigational pharmacy log. If for some reason any vials of Ad-sig-ecdhMUC-1/ecdCD40L vector are thawed but not administered to a patient the vial/vials will be destroyed according to standard biohazard waste disposal guidelines.

9.3 Vaccine dose and schedule

Eligible subjects who have signed an informed consent will receive the vector subcutaneously to the anterior thigh, shoulder or forearm at the scheduled visits. Vaccination will be carried out at NCC's Ambulatory Treatment Unit (ATU). Paracetamol 1 gram will be given as premedication approximately 15-30 minutes before vaccination. The subject will be monitored for at least 2 hours after the injection. Blood pressure, pulse rate, oxygen saturations and temperature will be recorded at 15 minute intervals. ECG will be performed before vaccination and 2 hours post vaccination. If the vital signs are not stable, or if the patient is complaining of rigors or other side effects such as fever or hypotension (systolic below 90 mm mercury), the physician will be immediately notified.

Appropriate dose modifications for the Ad-sig-hMUC-1/ecdCD40L vector are outlined below in Table 2. No new investigational or commercial agents or therapies other than those described in this protocol (and other than protocol-allowed ongoing cancer treatment that patient started prior to entry in this trial) may be administered with the intent to treat the patient's malignancy during the treatment period of this trial. However, if a patient develops progressive disease requiring urgent treatment with conventional cytotoxic chemotherapy, they will be allowed to come off protocol to seek such therapies.

Table 2 Dose Levels and Schedules for Ad-sig-hMUC-1/ecdCD40L

Cohorts	Dose in Viral Particles (Injection Day)	Total Dose in Viral Particles (Per Patient)
One	$1x10^9$ VP	$1x10^9$ VP
Two	$1x10^{10}$ VP	$1x10^{10}$ VP
Three	$5x10^{10}$ VP	$5x10^{10}$ VP
Four	$1x10^{11}$ VP	$1x10^{11}$ VP
Five	$1x10^{11}$ VP Days 1 and 8	$2x10^{11}$ VP
Six	$1x10^{11}$VP Days 1, 8 and 22	$3x10^{11}$ VP
Seven	$1x10^{11}$VP Days 1, 8, 22, 52, and 82	$5x10^{11}$ VP

10. DRUG SUPPLIES AND PREPARATION

10.1 Vector Production

(Redacted)

10.2 Viral Vector Particle Supply

(Redacted)

10.3 Viral Vector Dilution

The vials were filled with 0.6cc per vial to allow an effective dose of 0.5 cc to be delivered minus the drug in the syringe head and what is left in the vial.

The following supplies will be furnished

- Vials of drug in two concentrations, $5x10^9$ and $5x10^{10}$ vector particles per 0.5cc.
- All dispensed or disposed of drug and supplies used for handling drug are to be handled as biohazardous waste and only disposed of in containers or bags so labeled.

Instructions for Dose for Cohort Number 1
You will use part of the contents from the vial labeled *$5x10^9$* vp
Step 1.
Remove one vial labeled $5x10^9$ vp from freezer and place upright in a rack.
Allow to thaw at room temperature for approximately 10 minutes.

If the contents of the vial has not completely thawed by the end of 10 minutes, then the vial is held in contact with the skin of the hand and gently swirled without frothing until completely thawed.

Step 2.

Swirl vial to mix contents. Spray vial of vector particles and vial of viral diluent with 70% alcohol and place in rack in BSL2 biosafety cabinet.

Step 3.

Using insulin 1cc syringe with a 26 or 28 gauge needle. Draw up 0.1cc from vial under hood. Cap syringe.

Record patient information and date and time of dispensing and dispensing volume on the dispensing label and paste the label on the syringe.

Step 4.

Place syringe in sealed plastic bag and place the sealed bag in a box with ice-pack. Deliver the box to ATU where study treatment is being given.

Dispose vial with remaining drug in a Biohazard waste bag or container.

Record in vial disposal sheet with patient information and date and time of dispensing.

Record drug accountability log accordingly and paste the used dispensing label on the log too.

Instructions for Dose for Cohort Number 2

You will use the contents from two vials labeled *$5x10^9$* vp.

Step 1.

Remove 2 vials labeled $5x10^9$ vp from freezer and upright in a rack.

Allow to thaw at room temperature for approximately 10 minutes.

If the contents of the vial has not completely thawed by the end of 10 minutes, then the vial is held in contact with the skin of the hand and gently swirled without frothing until completely thawed.

Step 2.

Swirl vial to mix contents. Spray vial with 70% alcohol and place in rack in BSL2 biosafety cabinet.

Step 3.

Using insulin 1cc syringe with a 26 or 28 gauge needle. Draw up 0.5cc from each vial into same syringe under hood (total 1.0cc). Cap syringe.

Record patient information and date and time of dispensing and dispensing volume on the dispensing label and paste the label on the syringe.

Step 4.

Place syringe in sealed plastic bag and place the sealed bag in a box with ice-pack. Deliver the box to ATU where study treatment is being given.

Dispose vial with remaining drug in a Biohazard waste bag or container.

Record in vial disposal sheet with patient information and date and time of dispensing.

Record drug accountability log accordingly and paste the used dispensing label on the log too.

Instructions for Dose for Cohort Number 3

You will use the contents from one vial labeled *$5x10^{10}$* vp.

Step 1.

Remove one vial labeled $5x10^{10}$ VP from freezer and upright in a rack.

Allow to thaw at room temperature for approximately 10 minutes.

If the contents of the vial has not completely thawed by the end of 10 minutes, then the vial is held in contact with the skin of the hand and gently swirled without frothing until completely thawed.

Step 2.

Swirl vial to mix contents. Spray vial with 70% alcohol and place in rack in BSL2 biosafety cabinet.

Step 3.

Using insulin 1cc syringe with a 26 or 28 gauge needle. Draw up 0.5cc from vial under hood. Cap syringe.

Record patient information and date and time of dispensing and dispensing volume on the dispensing label and paste the label on the syringe.

Step 4.

Place syringe in sealed plastic bag and place the sealed bag in a box with ice-pack. Deliver the box to ATU where study treatment is being given.

Dispose vial with remaining drug in a Biohazard waste bag or container.
Record in vial disposal sheet with patient information and date and time of dispensing.
Record drug accountability log accordingly and paste the used dispensing label on the log too.

Instructions for Dose for Cohort Number 4
You will use the contents from two vials labeled *$5x10^{10}$* vp.
Step 1.
Remove 2 vials labeled $5x10^{10}$ vp from freezer and place upright in a rack.
Allow to thaw at room temperature for approximately 10 minutes.
If the contents of the vial has not completely thawed by the end of 10 minutes, then the vial is held in contact with the skin of the hand and gently swirled without frothing until completely thawed.
Step 2.
Swirl the 2 vials to mix contents. Spray vial with 70% alcohol and place in rack in BSL2 biosafety cabinet.
Step 3.
Using insulin 1cc syringe with a 26 or 28 gauge needle. Draw up 0.5cc from each vial into same syringe under hood (total 1.0cc). Cap syringe.
Record patient information and date and time of dispensing and dispensing volume on the dispensing label and paste the label on the syringe.
Step 4.
Place syringe in sealed plastic bag and place the sealed bag in a box with ice-pack. Deliver the box to ATU where study treatment is being given.
Dispose vial with remaining drug in a Biohazard waste bag or container.
Record in vial disposal sheet with patient information and date and time of dispensing.
Record drug accountability log accordingly and paste the used dispensing label on the log too.

Instructions for Dose for Cohort Number 5 and 6
For each of the injections, you will use the contents from two vials labeled *$5x10^{10}$* vp.
Step 1.
Remove 2 vials labeled $5x10^{10}$ vp from freezer and place upright in a rack.
Allow to thaw at room temperature for approximately 10 minutes.
If the contents of the vial has not completely thawed by the end of 10 minutes, then the vial is held in contact with the skin of the hand and gently swirled without frothing until completely thawed.
Step 2.
Swirl the 2 vials to mix contents. Spray vial with 70% alcohol and place in rack in BSL2 biosafety cabinet.
Step 3.
Using insulin 1cc syringe with a 26 or 28 gauge needle. Draw up 0.5cc from each vial into same syringe under hood (total 1.0 cc). Cap syringe.
Record patient information and date and time of dispensing and dispensing volume on the dispensing label and paste the label on the syringe.
Step 4.
Place syringe in sealed plastic bag and place the sealed bag in a box with ice-pack. Deliver the box to ATU where study treatment is being given.
Dispose vial with remaining drug in a Biohazard waste bag or container.
Record in vial disposal sheet with patient information and date and time of dispensing.
Record drug accountability log accordingly and paste the used dispensing label on the log too.

Instructions for Dose for Cohort Number 5 and 6 receiving 2 fold dose reduction vaccination

For each of the injections, you will use the contents from one vial labeled *$5x10^{10}$* vp.
Step 1.
Remove 1 vial labeled $5x10^{10}$ vp from freezer and place upright in a rack.
Allow to thaw at room temperature for approximately 10 minutes. . If the contents of the vial has not completely thawed by the end of 10 minutes, then the vial is held in contact with the skin of the hand and gently swirled without frothing until completely thawed..

Step 2.
Swirl the vial to mix contents. Spray vial with 70% alcohol and place in rack in BSL2 biosafety cabinet.
Step 3.
Using insulin 1cc syringe with a 26 or 28 gauge needle. Draw up 0.5cc from the vial into same syringe under hood (total 0.5 cc). Cap syringe.
Record patient information and date and time of dispensing and dispensing volume on the dispensing label and paste the label on the syringe.
Step 4.
Place syringe in sealed plastic bag and place the sealed bag in a box with ice-pack. Deliver the box to ATU where study treatment is being given.
Dispose vial with remaining drug in a Biohazard waste bag or container.
Record in vial disposal sheet with patient information and date and time of dispensing.
Record drug accountability log accordingly and paste the used dispensing label on the log too.

Instructions for Dose for Cohort Number 5 and 6 receiving 4 fold dose reduction vaccination

For each of the injections, you will use the contents from one vial labeled $5x10^{10}$ vp.
Step 1.
Remove 1 vial labeled $5x10^{10}$ vp from freezer and place upright in a rack.
Allow to thaw at room temperature for approximately 10 minutes. . If the contents of the vial has not completely thawed by the end of 10 minutes, then the vial is held in contact with the skin of the hand and gently swirled without frothing until completely thawed..
Step 2.
Swirl the vial to mix contents. Spray vial with 70% alcohol and place in rack in BSL2 biosafety cabinet.
Step 3.
Using insulin 1cc syringe with a 26 or 28 gauge needle. Draw up 0.25cc from the vial into same syringe under hood (total 0.25 cc). Cap syringe.
Record patient information and date and time of dispensing and dispensing volume on the dispensing label and paste the label on the syringe.
Step 4.
Place syringe in sealed plastic bag and place the sealed bag in a box with ice-pack. Deliver the box to ATU where study treatment is being given.
Dispose vial with remaining drug in a Biohazard waste bag or container.
Record in vial disposal sheet with patient information and date and time of dispensing.
Record drug accountability log accordingly and paste the used dispensing label on the log too.

Instructions for Dose for Cohort Number 7

For each of the injections, you will use the contents from two vials labeled $5x10^{10}$ vp.
Step 1.
Remove 2 vials labeled $5x10^{10}$ vp from freezer and place upright in a rack.
Allow to thaw at room temperature for approximately 10 minutes.
If the contents of the vial has not completely thawed by the end of 10 minutes, then the vial is held in contact with the skin of the hand and gently swirled without frothing until completely thawed.
Step 2.
Swirl the 2 vials to mix contents. Spray vial with 70% alcohol and place in rack in BSL2 biosafety cabinet.
Step 3.
Using insulin 1cc syringe with a 26 or 28, gauge needle. Draw up 0.5cc from each vial into same syringe under hood (total 1.0 cc). Cap syringe.
Record patient information and date and time of dispensing and dispensing volume on the dispensing label and paste the label on the syringe.
Step 4.
Place syringe in sealed plastic bag and place the sealed bag in a box with ice-pack. Deliver the box to ATU where study treatment is being given.
Dispose vial with remaining drug in a Biohazard waste bag or container.

Record in vial disposal sheet with patient information and date and time of dispensing.
Record drug accountability log accordingly and paste the used dispensing label on the log too.

10.4 SOP for handling a vector vaccine preparation spill

a) An adsorbent paper will be placed on the puddle of the spill. This will be removed after all of the free liquid has been soaked into the paper. The paper will be placed into a biohazard bag.
b) Then, the surface that was contaminated will be wiped with or 10% bleach solution from the outside in. The papers used for the cleaning will be placed into the biohazard bag.
If the patient uses the bathroom at the site on the date of the vector injection, the toilet will be flushed with 10% bleach solution in compliance with clinic policy.

11. SUBJECT SAFETY

11.1 Adenoviral vaccine toxicities

Adenoviral vectors are the most popular delivery system for gene therapy of cancer, having been tested in clinical trials involving more than 400 patients. Clinical data has been generated mainly for replication-incompetent vectors containing the p53 gene, in trials that were designed to restore the tumor suppressor function of the p53 gene in patients carrying a mutated form of this gene. Such trials[37-44] showed that doses of the adenoviral vector ranging from $9x10^9$ to $7.5x10^{12}$ vector particles (VP) could be injected intratumorally repeatedly into patients without causing dose-limiting hypersensitivity or other toxicity. Multivariate analysis of vector related parameters, including dose, route of injection, type of transgene and number of injections, indicated that none of these parameters were reliable predictors of adverse events. The conclusion from these studies was that the toxicities observed (fevers, chills, rash, swelling at injection site, nausea, headache, dizziness, fatigue, difficulty breathing, transient elevation of white cell count, lactate dehydrogenase, serum transaminases, prothrombin time, and transient lowering of platelet count, transient lowering of wbc and platelet count, and transient increases in IL6, IL10 and TNF alpha) were related to patient factors rather than vector factors, including the patient's age and underlying symptoms and the inclusion of surgery in the trial.[46-47]

The problem of toxicity associated with adenoviral vectors was reviewed in an NIH Recombinant DNA Advisory Committee meeting on December 8-9, l999, following the death of a patient undergoing treatment for ornithine transcarbamylase (OTC) deficiency at the University of Pennsylvania. This patient had been injected with $2.5x10^{12}$ replication-incompetent adenoviral vector particles directly into the hepatic artery, resulting in high fevers, disseminated intravascular coagulation, acute respiratory distress syndrome, acute hepatic, cardiac and renal failure, anemia, coma, thrombocytopenia, conjugated hyperbilirubinemia, and finally death within 72 hours. Toxicity was associated with high levels of TNF-alpha, IL6 and IL10 in the systemic circulation. Two factors could have contributed to the toxicity, namely injection of an exceptionally high number of adenovirus particles into the hepatic artery, and the OTC deficiency itself which results in impairment of the metabolic pathways mediating the conversion of ammonia, a toxic degradation product of protein metabolism, into urea. In an OTC-deficient patient, ammonia accumulates in the serum and tissues, and the increased levels of ammonia can lead to coma and death. The fever and tissue destruction associated with high levels of TNF-alpha, IL6 and IL10 dramatically increased the amount of ammonia produced in the patient.

Similar doses of replication-incompetent adenoviral vectors injected into patients who were not afflicted with OTC deficiency did not result in clinically significant toxicity.[46-47] Thus, most of the critical parameters in the University of Pennsylvania trial are not relevant to the protocol described in this proposal. Moreover, more than 400 patients have been injected intratumorally with replication-incompetent adenoviral vectors in other trials, and none of the patients developed clinically significant toxicity.[46-47] Since this is a Phase I trial and the Ad-sig-hMUC-

1/ecdCD40L vector has not been given to humans before there are no known risks associated with this agent. A list showing the possible short and long term adverse events specific for the Ad-sig-hMUC-1/ecdCD40L vector is found in Section 11.6.

11.2 Known Toxicity of CD 40 Ligand

Increased levels of soluble CD40L have been correlated with homocysteine levels and a risk of coronary artery disease. This may be related to the effect of the CD40L on macrophages and other cells with CD40 receptors in areas of inflammation on the luminal surface of endothelial cells. The injection of the Ad-sig-hMUC-1/ecdCD40L vector should not result in measurable levels of CD40L in the systemic circulation. No cardiac toxicity was seen in our pharmacologic toxicity studies with sc injection of the Ad-sig-hMUC-1/ecdCD40L vector. Others have injected CLL cells infected with the Ad-sig-CD40L vectors intravenously and there was no toxicity.[38] Fibroblasts infected with the Ad-sig-CD40L have been injected subcutaneously into human subjects and there were no significant adverse events.[37] Antibodies which are agonistic for the CD40 receptor have been injected into 244 patients without serious adverse events.[39-40] Finally, recombinant CD40L has been injected intravenously into human subjects without serious adverse events. [41] Please see extensive discussion of these clinical studies in Section 4.2 above.

11.3 Potential Ad-sig-hMUC-1/ecdCD40L Vector Specific Adverse Events

Short Term Complications: The patient could suffer from reduced blood pressure, elevated temperature, shortness of breath, depressed blood counts, local pain, rash and inflammation of the injection site and possible rigors. This list is based on information summarized from clinical trials in which adenoviral vectors were injected into tumor tissue or subcutaneously.[46-47]

Long Term Complications: Autoimmune disease. Although this is a theoretical possibility, experiments in chimps as well as three clinical studies in human subjects with MUC-1 vaccines suggest that this will not be a problem.[14, 16, 46]

11.4 Patient Protection

The Ethical Principles for Medical Research Involving Human Subjects outlined in the World Medical Association Declaration of Helsinki will be followed in the treatment of patients. The study will be carried out applying the principles of Good Clinical Practice. Patients who do not fulfill all of the eligibility requirements for the study will not be enrolled. Should there be any question regarding patient eligibility, the final decision on study entry is to be made by the principle investigator (PI) of the trial and MicroVAX. Subjects who are not able to comply with the requirements of the study will be removed from the study.

Patients with progressive disease will be offered the option for reassignment to other therapies, and will be taken off study. All patients that come off study will continue to be monitored for Serious Adverse Events for 30 days after removal or until resolution of the SAE.

11.5 Serious Adverse Event Definition

A serious adverse drug event is any adverse drug experience occurring at any dose that results in any of the following:

- Death
- A life-threatening adverse drug experience
- In-patient hospitalization or prolongation of existing hospitalization
- A persistent or significant disability/incapacity
- A congenital anomaly/birth defect

▪ Medical event that may jeopardize the patient and may require medical or surgical intervention to prevent one of the outcomes listed above

11.6 Serious Adverse Event Reporting

All serious adverse events will be reported to the Medical Monitor within 24 hours of finding out about the event. An SAE report form will be completed. For a reportable SAE, the Investigator (Dr Han Chong Toh) is responsible for submitting to the FDA within 7 calendar days a preliminary report with a complete report to follow within an additional 8 days for a total of 15 days. The DSMB will also be notified to assist in determining causality and will be involved in invoking stopping rules if indicated.

The Serious Adverse Event will also be reported by the Investigator to the Singhealth CIRB in accordance with CIRB guidelines.

11.7 Adverse Event Definition

An adverse event is any unfavorable, harmful, or unintended change involving function, structure, or chemistry change that occurs during the study, regardless of drug relationship, including any undercurrent illness, injury, toxicity, sensitivity, or sudden death.

A pre-existing condition is one that is present prior to or at the start of the study and is to be reported as part of the patient's medical history. It should be reported as an adverse event if the frequency, intensity, or the character of the condition worsens during study treatment.

Lack of efficacy is a worsening of the disease being studied or lack of desired effect of the study drug. If followed as an efficacy parameter, it should not be recorded as an adverse event.

11.8 Reporting Non-SAE Adverse Events

All adverse events that occur at any time during the study, including the 30 day post-treatment period as defined in the protocol after drug administration is discontinued, are to be reported in the patient's Case Report Forms. Any medications given to treat the event are recorded on the concomitant medications page.

The Investigator or Study Coordinator will evaluate each patient at each visit during the study for any new or continuing symptoms since the previous visit. Any symptoms changing in character or intensity should be noted. Any clinically significant adverse event reported by the patient or caregiver or noted by the investigator or study coordinator will be recorded on the Adverse Event Case Report Form. The intensity will be evaluated, relationship to study drug will be determined, and any necessary management will be recorded in the patient's medical record.

Not Related: The event does not meet the criteria above and the event is known to be associated with a clinical condition or with another medication taken by the patient.

Unknown: Current information about the event and the drug is insufficient to determine that the event is not related to the use of the drug. A definitive response will be required to determine the rehabilitation of the drug to the course of event.

A clinical laboratory abnormality should be reported as an adverse event only if the following conditions are met:

▪ The laboratory abnormality has been confirmed by at least one repeat test, or the abnormality suggests disease and/or organ toxicity, or
▪ The abnormality is of a degree that requires active management, e.g., change of dose, discontinuation of drug, more frequent follow-ups, diagnostic investigation.

Health hazard or side effect should also be reported as a Serious Adverse Event.

"Death" should not be reported as an adverse event. The cause of death should be reported as an adverse event. The only exception is "Sudden Death" when the cause is unknown.

11.9 Toxicity Grading

All toxicities will be evaluated and graded according to the NCI Common Toxicity Criteria Adverse Events Version 4. (http://evs.nci.nih.gov/ftp1/CTCAE/CTCAE_4.03_2010-06-14_Quick Reference_5x7.pdf)

11.10 Criteria For Removal From Study

Patients may be removed from the study for the following reasons:

- Adverse Events: Any adverse event that does not resolve or that interferes with patient compliance
- Rapid Disease Progression: Patients will be taken off study if they have rapidly progressive disease per investigator's discretion.
- Personal Reasons: As stated in the informed consent, patients may withdraw from the study at any time for any reason.
- Clinical Judgment of the Investigator: A patient may be withdrawn from the study if, in the opinion of the investigator, it is not in the patient's best interest to continue (adverse event, concurrent illness, etc).

12. PATIENT EVALUATION

12.1 Study Calendar

The Calendar Schedule for the patient evaluation is provided below in Tables 3A, 3B, 3C and 3D.

Table 3A: Study Calendar for Cohorts 1-4 of Ad-sig-hMUC-1/ecdCD40L Vector Vaccine Study

	Screening	Visit 1	Visit 2 (±6 hours)	Visit 3 (±6 hours)	Visit 4 (±3 days)	Visit 5 (±7 days)	Visit 6 (±7 days)	Visit 7 (±7 days)	Follow-up
**Procedures**	**Prior to Study Entry (≤ 28 days)**	**Day 1** Vector injection	**Day 2**	**Day 3**	**Day 15**	**Week 5**	**Week 9**	**Week 13**	**Every 12 weeks (+/- 7 days)[H]**
Signed Consent Form	X								
History	X	X							
Physical Exam	X	X	X	X	X	X	X	X	
Vital signs	X	X	X	X	X	X	X	X	
Echocardiogram/MUGA	X								
Chest X ray/Other radiographs[A]	X								
ECG	X	X		X	X	X	X	X	
Toxicity Evaluation		X	X	X	X	X	X	X	
Prior/Concomitant medications	X	X	X	X	X	X	X	X	
Staging scan	X						X[I]		X[F]
Hematology[B]	X	X[E]		X	X	X	X	X	
Biochemistry [C]	X	X[E]		X	X	X	X	X	
Pregnancy Test	X								
Infectious disease test[D]	X								
CA 27-29 or CA15-3 (MUC-1)	X					X	X	X	
HLA typing		X[E]							
20mL Research bloods (IL-6, IL-10, TNFα)			X	X					
40mL Research bloods		X[E]				X	X	X	
Anticancer therapy and survival assessme									X[G]

Note: The FDA has agreed that the schedule for some of the tests could change slightly after analyzing the tests from the first few subjects.

A: If clinically indicated

B: Hematology (Full Blood counts)

C: Biochemistry:

- Renal panel: urea, sodium, potassium, chloride, bicarbonate, creatinine and glucose
- Liver function panel: total protein, albumin, total bilirubin, alkaline phosphatase, AST, ALT
- calcium, magnesium, phosphate

D: HIV, Hepatitis BsAg, anti-HCV antibody, VDRL (if not done in the past one year)

E: pre-injection

F: Review of tumor assessment if performed as per institutional standard

G: Information about survival and subsequent anti-cancer therapies will be collected via telephone calls, patient medical records and or clinic visits approximately every 12 weeks until death, loss to follow-up or study termination unless the patient requests to be withdrawn from follow-up.

H: Every 12 weeks from last visit

I: Staging CT scans 2 months after last vaccination with a window period of ±7 days.

Table 3B: Study Calendar for Cohort 5 of Ad-sig-hMUC-1/ecdCD40L Vector Vaccine Study

	Screening	Visit 1	Visits2 and 3 (±6 hours)	Visit 4 (±6 hours)	Visits 5 and 6 (±6 hours)	Visit 7 (±3 days)	Visits 8, 9 and 10 (±7 days)	Follow-up
**Procedures**	**Prior to Study Entry (≤ 28 days)**	**Day 1** Vector Injection #1	**Days 2 and 3**	**Day 8** Vector Injection #2	**Days 9 and 10**	**Day 22**	**Week 6, 10, 14**	**Every 12 weeks (+/- 7 days)[J]**
**Signed Consent Form**	X							
**History**	X	X						
**Physical Exam**	X	X	X	X	X	X	X	
**Vital signs**	X	X	X	X	X	X	X	
**Echocardiogram/MUGA**	X							
**Chest X ray/Other radiographs[A]**	X							
**ECG**	X	X	X[F]	X	X[G]	X	X	
**Toxicity Evaluation**		X	X	X	X	X	X	
**Prior/Concomitant medications**	X	X	X	X	X	X	X	
**Staging scan**	X						X[K]	X[H]
**Hematology[B]**	X	X[E]	X[F]	X	X[G]	X	X	
**Biochemistry [C]**	X	X[E]	X[F]	X	X[G]	X	X	
**Pregnancy Test**	X	X[E]						
**Infectious disease test[D]**	X							
**CA 27-29 or CA15-3 (MUC-1)**	X						X	
**HLA typing**		X[E]						
**20mL Research bloods (IL-6, IL-10, TNFα)**			X		X			
**40mL Research bloods**		X[E]				X	X	
**Anticancer therapy and survival assessment**								X[I]

Note: The FDA has agreed that the schedule for some of the tests could change slightly after analyzing the tests from the first few subjects.

A: If clinically indicated

B: Hematology (Full Blood counts)

C: Biochemistry:

- Renal panel: urea, sodium, potassium, chloride, bicarbonte, creatinine and glucose
- Liver function panel: total protein, albumin, total bilirubin, alkaline phosphatase, AST, ALT

- calcium, magnesium, phosphate

D: HIV, Hepatitis BsAg, anti-HCV antibody, VDRL (if not done in the past one year)

E: pre-injection

F: Assays and evaluations carried out on Day 3 only

G: Assays and evaluations carried out on Day 10 only

H: Review of tumor assessment if performed as per institutional standard

I: Information about survival and subsequent anti-cancer therapies will be collected via telephone calls, patient medical records and or clinic visits approximately every 12 weeks until death, loss to follow-up or study termination unless the patient requests to be withdrawn from follow-up.

J: Every 12 weeks from last visit

K: Staging CT scans 2 months after last vaccination with a window period of ±7 days.

Table 3C: Study Calendar for Cohort 6 of Ad-sig-hMUC-1/ecdCD40L Vector Vaccine Study

	Screening	Visit 1	Visits2 and 3 (±6 hours)	Visit 4 (±6 hours)	Visits 5 and 6 (±6 hours)	Visit 7 (±6 hours)	Visits 8-9 (±6 hours)	Visit 10 (+3 days)	Visits 11, 12 and 13 (±7 days)	Follow-up
**Procedures**	**Prior to Study Entry (≤ 28 days)**	**Day 1** Vector Injection #1	**Days 2 and 3**	**Day 8** Vector Injection #2	**Days 9 and 10**	**Day 22** Vector Injection #3	**Days 23 and 24**	**Day 36**	**Week 8, 12, 16**	**Every 12 weeks (+/- 7 days)[K]**
**Signed Consent Form**	X									
**History**	X	X								
**Physical Exam**	X	X	X	X	X	X	X	X	X	
**Vital signs**	X	X	X	X	X	X	X	X	X	
**Echocardiogram**	X									
**Chest X ray/Other radiographs[A]**	X									
**ECG**	X	X	X[F]	X	X[G]	X	X[H]	X	X	
**Toxicity Evaluation**		X	X	X	X	X	X	X	X	
**Prior/Concomitant medications**	X	X	X	X	X	X	X	X	X	
**Staging scan**	X								X[L]	X[I]
**Hematology[B]**	X	X[E]	X[F]	X	X[G]	X	X[H]	X	X	
**Biochemistry [C]**	X	X[E]	X[F]	X	X[G]	X	X[H]	X	X	
**Pregnancy Test**	X	X[E]								
**Infectious disease test[D]**	X									
**CA 27-29 or CA15-3 (MUC-1)**	X								X	
**HLA typing**		X[E]								
**20mL Research bloods (IL-6, IL-10, TNFα)**			X		X		X			
**40mL Research bloods**		X[E]						X	X	
**Anticancer therapy and survival assessment**										X[J]

Note: The FDA has agreed that the schedule for some of the tests could change slightly after analyzing the tests from the first few subjects.

A: If clinically indicated

B: Hematology (Full Blood counts)

C: Biochemistry:

- Renal panel: urea, sodium, potassium, chloride, bicarbonte, creatinine and glucose

- Liver function panel: total protein, albumin, total bilirubin, alkaline phosphatase, AST, ALT
- calcium, magnesium, phosphate

D: HIV, Hepatitis BsAg, anti-HCV antibody, VDRL (if not done in the past one year)

E: pre-injection

F: Assays and evaluations carried out on Day 3 only

G: Assays and evaluations carried out on Day 10 only

H: Assays and evaluations carried out on Day 24 only

I: Review of tumor assessment if performed as per institutional standard

J: Information about survival and subsequent anti-cancer therapies will be collected via telephone calls, patient medical records and or clinic visits approximately every 12 weeks until death, loss to follow-up or study termination unless the patient requests to be withdrawn from follow-up.

K: Every 12 weeks from last visit

L: Staging CT scans 2 months after last vaccination with a window period of ±7 days.

Table 3D: Study Calendar for Cohort 7 of Ad-sig-hMUC-1/ecdCD40L Vector Vaccine Study

	Screening	Visit 1	Visits2 and 3 (±6 hours)	Visit 4 (±6 hours)	Visits 5 and 6 (±6 hours)	Visit 7 (±6 hours)	Visits 8-9 (±6 hours)	Visit 10-11 (±3days)	Visits 12-13 (±7 days)	Follow-up
Procedures	Prior to Study Entry (≤ 28 days)	Day 1 Vector Injection #1	Days 2 and 3	Day 8 Vector Injection #2	Days 9 and 10	Day 22 Vector Injection #3	Days 23 and 24	Days 52 and 82 Vector Injection #4-5	Follow up Visits Days 112 and 172	Every 12 weeks (+/- 7 days)[O]
Signed Consent Form	X									
History	X	X								
Physical Exam	X	X	X	X	X	X	X	X	X	
Vital signs	X	X	X	X	X	X	X	X	X	
Echocardiogram	X									
Chest X ray/Other radiographs[A]	X									
ECG	X	X[F]	X[G]	X[F]	X[H]	X[F]	X[I]	X[F]	X	
Toxicity Evaluation		X	X	X	X	X	X	X	X	
Prior/Concomitant medications	X	X	X	X	X	X	X	X	X	
Staging scan	X							X[J]	X[J]	X[M]
Hematology[B]	X	X[F]	X[G]	X[F]	X[H]	X[F]	X[I]	X[F]	X	
Biochemistry	X[C]	X[C, F]	X[D, G]	X[C, F]	X[D, H]	X[C, F]	X[D, I]	X[D, F]	X[D]	
Pregnancy Test	X	X[F]								
Infectious disease test[E]	X									
CA 27-29 or CA15-3 (MUC-1)	X							X[F]	X	
HLA typing		X[F]								
20mL Research bloods (IL-6, IL-1 TNFα)[N]			X		X		X			
40mL Research bloods		X[F]						X[F, K]	X[K]	
Anticancer therapy and survival assessment										X[N]

Note: The FDA has agreed that the schedule for some of the tests could change slightly after analyzing the tests from the first few subjects.

A: If clinically indicated;

B: Hematology (Full Blood counts)

C: Biochemistry:

- Renal panel: urea, sodium, potassium, chloride, bicarbonate, creatinine and glucose
- Liver function panel: total protein, albumin, total bilirubin, alkaline phosphatase, AST, ALT
- calcium, magnesium, phosphate

D: Biochemistry:

- Renal panel: urea, sodium, potassium, chloride, bicarbonate, creatinine and glucose

- Liver function panel: total protein, albumin, total bilirubin, alkaline phosphatase, AST, ALT

E: HIV, Hepatitis BsAg, anti-HCV antibody, VDRL (if not done in the past one year);

F: pre-injection (-1 day window)

G: Assays and evaluations carried out on Day 3 only;

H: Assays and evaluations carried out on Day 10 only;

I: Assays and evaluations carried out on Day 24 only

J: Staging CT scans on days 52, 112, 172 with a window period of ±7 days.

K: 40mL research bloods on Days 52, 112 and 172

L: If patient of cohort 7 develops signs of immune reaction post vaccination (from 4th to 5th vaccine injection), post-injection IL-6, IL-10 and TNFα will be collected during next scheduled visit.

M: Review of tumor assessment if performed as per institutional standard

N: Information about survival and subsequent anti-cancer therapies will be collected via telephone calls, patient medical records and or clinic visits approximately every 12 weeks until death, loss to follow-up or study termination unless the patient requests to be withdrawn from follow-up.

O: Every 12 weeks from last visit

12.2 Baseline / Pre-immunization (not more than 28 days prior to first vaccination)

12.2.1 History. A complete history with full details of the patient's previous treatment and response will be obtained, including:

- Patient exposure to steroids, radiation and chemotherapy drugs (total dosage of each drug and when given).
- Previous or current fever, infections and antibiotic treatment.
- Previous CNS involvement
- Clinical picture at initial presentation including ECOG score.
- Prior immunologic and cytogenetic studies of the patients' tumor cells.

12.2.2 Clinical Evaluation (all measurements in metric units):

- A complete physical examination.
- Vital signs including height, weight, blood pressure, pulse rate, oxygen saturation and temperature
- Chest and other radiographs as clinically indicated.
- Documentation of tumour status prior to accrual into the clinical trial

12.2.3 Screening Investigations*:*

- Low resolution HLA typing (if not done before)
- FBC, renal function test, liver function test, Mg2+, Ca2+, PO4-,
- CA15-3
- HIV, Hepatitis BsAg, anti-HCV antibody, VDRL (if not done in the past one year)
- 2 dimensional ECHO or MUGA (if not done in the past one year)
- ECG
- Serum Pregnancy Test (for subject with child-bearing potential)

12.2.4 Baseline Scan Evaluation.

Baseline CT/MRI/PET scans of involved sites prior to first injection of vaccine

12.2.5 Baseline Research Bloods

Up to 40 millilitres of research blood will be taken prior to first SC injection of Ad-sig-hMUC1/ecdCD40L, collected in EDTA tubes for immune analysis which will include the following:

- Flow cytometric analysis for Immune subpopulations
- Baseline soluble TNFa, IL-6 and IL10 levels by ELISA assay
- Others (i.e., Pentamer assays, Cytokine Multiplex assays)

12.3 Pre-vaccination

- Patients will be examined by a physician prior to vaccination.
- Vital signs including blood pressure, pulse rate, oxygen saturation, and temperature will be recorded prior to injection.
- Patients will also be evaluated for central nervous system, respiratory, cardiac, abdominal and skin signs and symptoms
- FBC, renal function test, liver function test and ECG will be performed prior to vaccination (-1 day window)
- Mg^{2+}, Ca^{2+} and PO^{4-} will be performed prior to vaccination (only for Day 8 of Cohort 5-7 and Day 22 of Cohort 6-7) (-1 day window)

For cohort 7 (±3 days)

- CA15-3 (only for Days 52 and 82)
- 40mLs Research bloods will be taken on Day 52
- CT/MRI/PET scan of involved areas will be performed on Day 52 (± 7 days)

12.4 Post vaccination – 24 and 48 hours clinical review, blood and cytokine analysis (±6 hours) _(not applicable for 4th to 5th injections of Cohort 7)_

- Patients will be examined by a physician approximately 24 hours and 48 hours in Cohorts 1-7 (in all the cohorts) after each vaccination, except for the 4th and 5th injections of Cohort 7.
- Vital signs including blood pressure, pulse rate, oxygen saturation, and temperature will be recorded at each review
- Patients will also be evaluated for central nervous system, respiratory, cardiac, abdominal and skin signs and symptoms
- Local injection site will be examined and significant findings documented
- Soluble TNFa, IL-6 and IL10 levels will be drawn 24 hours and 48 hours after each vaccine injection
- FBC, renal function test, liver function test and ECG will be performed at approximately 48 hrs after each vaccination
- If patient of cohort 7 develops signs of immune reaction post vaccination from 4th to 5th vaccine injection, post-injection IL-6, IL-10 and TNFα will be collected during next scheduled visit.

12.5 Post Vaccine Evaluation – Day 15 (±3days) (cohorts 1-6)

- Patients will be evaluated on Day 15 post last vaccination (Day 15 in cohorts 1-4, Day 22 in cohort 5 and Day 36 in cohort 6).
- Vital signs including blood pressure, pulse rate, oxygen saturation, and temperature will be recorded
- Patients will also be evaluated for central nervous system, respiratory, cardiac, abdominal and skin signs and symptoms
- Local injection site will be examined and significant findings documented
- FBC, renal function test, liver function test and ECG will be performed prior to review

12.6 Post Vaccine Evaluation – Month 1, 2 and 3 (±7 days) (cohorts 1-6)

- Patients evaluated Month 1, 2 and 3 post last vaccination (Week 5, 9 and 13 for cohorts 1-4, Week 6, 10 and 14 for cohort 5, Week 8, 12 and 16 for cohort 6).
- Vital signs including blood pressure, pulse rate, oxygen saturation, and temperature will be recorded
- Patients will also be evaluated for central nervous system, respiratory, cardiac, abdominal and skin signs and symptoms
- FBC, renal function test, liver function test and ECG will be performed at or just before each monthly clinic evaluation
- CA15-3 will be performed at or before each monthly clinic evaluation
- 40 mLs Research bloods will be taken
- CT/MRI/PET scan of involved areas will be performed just prior to review on Month 2 after the last vaccination
- Further Evaluation CT/MRI/PET scans will be performed according to investigator discretion

12.7 Post Vaccine Evaluation – Follow-up visits (±7 days) (cohort 7)

- Patients will be evaluated in Cohort 7 on Days 112 and 172.
- Vital signs including blood pressure, pulse rate, oxygen saturation, and temperature will be recorded
- Patients will also be evaluated for central nervous system, respiratory, cardiac, abdominal and skin signs and symptoms
- FBC, renal function test, liver function test and ECG will be performed at or just before each monthly clinic evaluation
- CA15-3 will be performed at or before each monthly clinic evaluation

- 40mLs Research bloods will be taken on Days 112 and 172.
- CT/MRI/PET scan of involved areas will be performed on Days 112 and 172.
- Further Evaluation CT/MRI/PET scans will be performed according to investigator discretion

12.8 Post Vaccine Evaluation – Follow-up visits (±7 days) (cohorts 1-7)

- Patients will be followed for initiation of new anticancer therapies, tumor assessment (on CT or MRI scans performed as per standard of care) and survival through telephone calls, patient medical records or clinic visits

13. RESPONSE EVALUATION

This trial is not designed to determine clinical efficacy. Subjects may not have measurable disease by RECIST criteria (eg good partial response or complete response following therapy) and thus may not be evaluable for clinical response by imaging.

If a patient's serum MUC-1 (ie CA15-3) levels decrease by more than 50 units and remain at that level or lower for four weeks after the vaccination, it will be concluded that the vaccine has *potential* efficacy.

Patients of cohorts 1-6 will have a CT/MRI/PET scan examination at baseline and month 2 follow-up visit. Patients of cohort 7 will have a CT/MRI/PET scan examination at baseline, and Days 52, 112, 172, 232, 292, and 352. All subsequent imaging will be performed as per standard of care. Responses will be evaluated using RECIST criteria. Any Partial or Complete responses will be confirmed with a repeat scan 4 weeks later.

14. STUDY STOPPING RULES

A Data and Safety Monitoring Board (DSMB) will be established to monitor the trial.

The study will be terminated early in event of the following:

- Death attributable to study drug
- Any Grade 4 toxicity attributable to the study drug (specifically, any change in serum transaminase, blood pressure or respiratory rate or arterial saturation)
- At the discretion of the investigators (study investigators reserve the right to terminate the study or re-design the protocol at any time for any reason).
- Any Grade 3 toxicity associated with autoimmune disease

15. DATA MANAGEMENT

15.1 Data Management Team

(Redacted)

15.2 Study Documentation

(Redacted)

15.3 Monitoring and Audit

(Redacted)

15.4. Data Entry

(Redacted)

15.5 Data Archive

(Redacted)

16. ETHICAL AND REGULATORY STANDARDS

16.1 Ethical Principles

The trial will be conducted by the Clinical Trials and Epidemiological Sciences, National Cancer Centre Singapore. The office will conduct the trial according to the ICH GCP and HIPAA guidelines. This study will also be carried out in accordance with the World Medical Association Declaration of Helsinki (1964).

16.2 Patient Confidentiality

The personal data recorded on all documents will be regarded as confidential.

16.3 Withdrawal of Patients

The patient can decide to withdraw from the study at any time for any reason. The Principal Investigator also has the right to withdraw the patient from the study if he decides that it is in the best interests of the patient. Full details of the reasons for withdrawal should be recorded in the case notes and on the clinical research forms. Withdrawn patients should be followed up in accordance with the protocol unless the patient withdraws consent.

Rapid clinical or radiological progression of disease will result in discontinuation from the study.

17. References

1. Jemal, A, Siegel, Ward E, et al. Cancer Statistics 2007, CA Cancer J Clin. 57(1):43-66. (2007).

2. Fossati R, Confalonieri C, Torri V, et al. Cytotoxic treatment for metastatic breast cancer. J Clin Oncol 16, 3439-3460, (1998).

3. Slamon DJ, Leyland-Jones B, Shak S, et al. Use of chemotherapy and monoclonal antibody Her-2-Neu for metastatic breast cancer. N Engl J Med 344(11), 783-92, (2001).

4. Starnes CO. Coley's toxins in perspective. Nature 357, 11-2, (1992).

5. Timmerman JM, Levy R. Cancer vaccines: pessimism in check. Nat Med 10, 1279 (2004); author reply 1279-80, (2004).

6. Rosenberg SA, Yang JC, Restifo NP. Cancer immunotherapy: moving beyond current vaccines. Nat Med 10,909-15, (2004).

7. DeVita, V, Hellman, S, and Rosenberg, S. Principles and Practice of Oncology. Lippincott, Philadelphia, 6th Edition, (1996).

8. Taylor-Papdiumitriou, J., Burchell, J., Miles, D.W., and Dalziel, M. MUC1 and Cancer. Biochim. Biophys. Acta. 1455, 301-313, (1999).

9. Koido, S., Kashiwaba, M., Chen, D.S., Gendler, S., Kufe, D., and Gong, J.L. Induction of antitumor immunity by vaccination of dendritic cells transfected with MUC1 Journal of Immunology 165: 5713-5719 (2000).

10. Jian Ren. Naoki Agata, Dongshu Chen, Yongaing Li, Wei-hsuan Lei Huang, Deepak Raina, Wen Chen, Surender Karbanda, and Donald Kufe. Human MUC1 carcinoma-associated protein confers resistance to genotoxic anticancer agents. Cancer Cell. 163-175 (2004).

11. Guddo F, Giatromanolaki A, Koukourakis, MI, Reina C, Bignola AM, Chlouverakis G, Hilkens J, Gatter KC, Harris, AL and Bopnsignore G. BUC1 expression in non-small cell lung cancer is independent of EGFR and c-erbB-2 expression and correlates with poor survival in node positive patients. J. Clin. Pathol. 51: 667-671, (1998).

12. Zhang S, Zhang HS, Reuter VE, Slovin SF, Scher HI, and Livingston PO. Expression of potential target antigens for immunotherapy on primary and metastatic prostate cancers. Clinical Cancer Research 4: 295-302, (1998).

13. Ajay P. Singh, Nicolas Moniaux, Sughash C. Chauhan, Jane L. Neza, an Surinder K Batra, Inhibition of MUC4 expression suppresses pancreatic tumor cell growth and metastasis, Cancer Research, 64: 622-630 (2004).

14. Muisselli C, Ragupathi G, Kilewski T, Panageas KS, Spinat Y, and Livingston PO. Reevaluation of the cellular immune response in breast cancer patients vaccinated with MUC1. Int. J. Cancer 97: 660-667, (2002).

15. Dong-Ming, Joh-Hwa Guh, Shih-Chieh Cheuh and Che-Ming Teng Modulation of anti-adhesion molecule MUC-1 is associated with arctin-induced growth inhibition in PC-3 cells. The Prostate 59:260-267 (2004).

16. Rochlitz C, Figlin R, Squiban P, Salzberg M, Pless M, Gerrmann R, Tartour E, Zhao YX, Bizouarne N, Baudin M, and Acres B. Phase I immunotherapy with a modified vaccinia virus (MVA) expressing human MUC1 as antigen-specific immunotherapy in patients with MUC1 positive advanced cancer. The Journal of Gene Medicine 5, 690-699, (2003).

17. Pantuck AJ, van Ophoven A, Bitlitz BJ, Tso CL, Acres B, Squiban P, Ross ME, Belldegrun AS, and Figlin RA. Journal of Immunotherapy 27, 240-253, (2004).

18. Rahn JJ, Dabbagh L, Pasdar M, Hugh JC. The importance of MUC-1 cellular localization I patients with breast carcinoma. Cancer: 91: 1973-1982, (2001).

19. Li US, Kanedo M, Sakamoto DG, Takeshima Y, and Inai K. The reverse apical pattern of MUC1 expression is characteristic of invasive micropapillary carcinoma of the breast. Breast Cancer 13: 58-63, (2006).

20. McGuckin MA, Walsh MD, Hohn BG, Ward BG, and Wrigtht RG. Prognositc sigtnificance of MUC1 epithelial mucin expression in breast cancer. Human Pathology 26: 432-439, (1995).

21. Kirschenbaum A, Itzkowitz SH, Wang JP, Yao S, Eliashvili M, and Levine AC. MUC1 expresion in prostate cancrinoma: correlation with grade and stage. Molecular Urology 3: 163-168, (l999).

22. Lapointe J., Li C, Higgins JP, van de Rihn M, Bair E, Montgomery K, Ferrari M, Egevad L, Rayford W, Bergerheim U, Eklman P, De Marzo AM, Tibshirani R, Botstein D, Brown PO, Brooks JD, and Pollack JR. Gene expression prolifling identifies clinically relevant subtypes of prostate cancer. Proc. National Acad. Sciences. 101: 811-816, (2004).

23. Giatromanoloki GF, Koukourakis MI, Reina C, Vignola AM, Chlouverakius G, Hilkens J, Gatter KC, Jarris AL and Bonsignore G. MUC1 expression in non-small cell lung cancer is independent of EGFR and c-erb-2 expression and correlates with poor survival in node positive patients. Journal Clinical Pathology 51: 667-671, (1998).

24. Nakamori S, Ota DM, Cleary KR, Shirotani K, and Irimura T. MUC1 mucin expression as a

marker of progression and metastasis of human colorectal carcinoma. Gastroenterology 106: 353-361, (1994).

25. Wang Li, Ma J, Liu FH, Yu QK, Chu GM, Perkins AC, and Li Y. Expression of MUC1 in primary and metastatic human epithelail ovarian cancer and its therapeutic significance. Gynecologic Oncology 105: 695-702, (2007).

26. Al-Hajj M., Wicha M., Benito-Hernandez A, Morrison SJ, and Clarke MF. Prospective identification of tumorigenic breast cancer cells. Proc. Natl. Acad. Sci. USA 100: 3983-3988, (2003).

27. Sperger JM, Chen X, Draper JS, Antosiewicz JE, Chon CH, Jones SB, Brooks JD, Andrews PW, Brown PO, and Tomson JA. Gene expression patterns in human embryonic stem cells and human pluripotent germ cell tumors. Proc. Natl. Acad. Sci. USA 100: 13350-13355, (2003).

28. Shipitsin M, Campbell LL, Argani P, Weremowicz S, Bloushtain-Qimron N, Yao J, Nikolskaya T, Serebryiskaya T, Beroukhim R, Hu M, Halushka MK, Sukumar S, Parker LM, Anderson KS, Harris LM, Garber JE, Richardson AL, Schnitt SJ, Nikolsky Y, Gelman RS, and Polyak Kornelia. Molecular definition of breast tumor heterogeneity. Cancer Cell 11: 259-273, (2007).

29. Zhang L, Tang YC, Akbulut H, Zelterman D, Linton PJ, and Deisseroth A. An adenoviral vector cancer vaccine that delivers a tumor-associated antigen/CD40L protein into dendritic cells. Proc. Natl. Acad. Sci. USA, 100, 15202-15106, (2003).

30. Tang Y, Zhang L, Yuan J, Akbulut H, Maynard J, Linton PJ, and Deisseroth A. Multistep process through which adenoviral vector vaccine overcomes anergy to tumor-associated antigens. Blood 104, 2704-2713 (2004).

31. Tang YC, Maynard J, Akbulut H, Petersen L, Fang XM, Zhang WW, Xia XQ, Koziol J, Linton PJ and Deisseroth A. Vector prime/protein boost vaccine which overcomes defects acquired during aging and cancer. Journal of Immunology, 177: 5697-5707, (2006).

32. Akbulut H, Tang YC, Akbulut KG, Maynard J, Zhang L, and Deisseroth A. Antitumor immune response induced by i.t. injection of vector-activated dendritic cells and chemotherapy suppresses metastatic breast cancer. Mole Cancer Ther 5:1975-1985, (2006)

33. Akbulut H, Akbulut KG, Tang YC, Maynard J and Deisseroth A. Chemotherapy targeted to cancer tissue potentiates antigen specific immune response induced by vaccine for in vivo antigen loading and activation of dendritic cells. Molecular Therapy, 10: 1753-1760, (2008).

34. Han TH, Tang, YC, Park YH, Petersen L, Maynard J, Li PC, and Deisseroth A. Ad-sig-BcrAbl/ecdCD40L vector prime-BcrAbl/ecdCD40L protein boost vaccine for P210Bcr-Abl protein, Bone Marrow Transplantation, 45: 550-557, (2010).

35. Tang, YC, Linton, PJ, Thoman M, and Deisseroth A. Symposium in Writing: vaccine for infections and cancer. Cancer Immunology and Immunotherapy 58: 1949-1957, (2009).

36. Deisseroth A, Tang Y, Zhang L, Akbulut H, and Habib N. TAA/ecdCD40L adenoviral prime-protein boost vaccine for cancer and infectious disease. Cancer Gene Therapy 20: 65-69, (2013).

37. Rousseau RF, Biagi E, Dutour A, Yvon ES, Brown MP, Lin T, Mei Z, Grillery B, Popek E, Heslop HE, Gee AP, Krance RA, Popai U, Carrum G, Margolin JR, and Brenner MK. Immunotherapy of high risk acute leukemia with a recipient (auotlogous) vaccine expressing transgenic human CD40L and IL-12 after chemotherapy and allogeneic stem cell transplnantation. Blood 107: 1332—1341, (2006).

38. Wierda WG, Cantwell JM, Woods SJ, Rassenti LZ, Prussak CE, and Kipps TJ. CD40L (CD154) gene therapy for CLL. Blood 96: 2917-2924, (2000).

39. Vonderheide RG et al. Clinical activity andimmmune moduleaiton in cancer patients treated with CO-870,893, a novel CD40 agonist monoclonal antibody. J. Clin. Oncology 25: 876-883, (2007).

40. Vonderhieide RH. Prospect of targeting the CD40 pathway for cancer therapy. Clinical Cancer Research 13: 1083-1088, (2007).

41. Vanderheidfe RH, Dutcher JP, Anderson JE, Eckhardt SG, Stephans KF, Razvillas B, Garl S, Butine MD, Perryh VP, Armitage RJ, Ghalie R, Caron DA, and Gribben JG. Phase I study of recombinant human CD40 ligand in cancer patiets. Journal of Clincial Oncology 19: 3280-3287, (2001).

42. Karpuses, M., Hsu, Y.M., Wang, J.H., Thompson, J., Lederman, S., Chess, L., and Thomas, D. Structure 3: 1031-1039 (1995).

43. Jefferson T, Rivetti D, Rivetti A, Rodin M, Di Pietrantonj C, and Demicheli V. Efficacy and effectiveness of influenza vaccines in elderly people: a systematic review. Lancet 366, 1165-1174, (2005).

44. Goodwin K, Viboud C, and Simonsen L. Antibody response to influenza vaccination in the elderly: a quantitative review. Vaccine 24, 1159-1169, (2006).

45. Dong L, More I, Hossain JM, Liu B, and Kimjra Y. An immunostimulatory oligodeoxynucleotide containing a cytosine-guanosine motif protects senescence-accelerated mice from lethal influenza virus by augmenting the T helper type 1 response. Journal of General Virology 84, 1623-1628, (2003).

46. Harvey, BGG, Maroni, J, O'Donoghue, KA, Chu, KW, Muscat, JC, Pippo, AL, Wright, CD, Hollmann, C, Wisnivesky, JP, Kessler, PD, Rasmussen, HS, Rosengart, TK, and Crystal, RG. Safety of local delivery of low-and intermediate-dose adenovirus gene transfer vectors to individuals with a spectrum of morbid conditions. Human Gene Therapy 13:15-63 (2002).

47. Crystal, RG, Harvey, BG, Wisnivesky, JP, O'Donoghue, KA, Chu, KW, Maroni, J, Muscat, JC, Pippo, AL, Wright, CE, Kaner, RJ, Leopold, PL, Kessler, Wang, B-Y, Chen, Y-B, Ayalon, O, Bender J, Analysis of risk factors for local delivery of low-and intermediate-dose of adenovirus gene transfer vectors to individuals with a spectrum of comorbid conditions. Human Gene Therapy 13:65-1000. (2002).

48. Dillman RO, Koziol JA. Phase I cancer trials: limitations and implications. Mol. Biother. 4:117-121, (1992).

49. Rosenberger WF, Haines LM. Competing designs for phase I clinical trials: a review. Statist. Med. 21:2757-2770, (2002).

50. Simon RM, Freidlin B, Rubinstein LV et al. Accelerated titration designs for phase I clinical trials in oncology. J. National Cancer Inst. 1138-1147, (1997).

51. Simon RM, Steinberg SM, Hamilton M et al. Clinical trial designs for the early clinical development of therapeutic cancer vaccines. J. Clin. Oncol. 19:1848-1854, (2001).

52. Storer BE. Design and analysis of phase I clinical trials. Biometrics 45:928-937 (1998).
