# Supplementary material for: A phase I study of an adenoviral vector delivering a MUC1/CD40-ligand fusion protein in patients with advanced adenocarcinoma
Source: Nat Commun. 2022 Oct 28;13:6453. doi: 10.1038/s41467-022-33834-4 (PMC9616917; doi:10.1038/s41467-022-33834-4)
Supplement: Supplementary file 3 — Reporting Summary [file 41467_2022_33834_MOESM3_ESM.pdf]

nature portfolio

Corresponding author(s): Salvatore Albani and Han Chong Toh

Last updated by author(s): Aug 1, 2022

Reporting Summary

Nature Portfolio wishes to improve the reproducibility of the work that we publish. This form provides structure for consistency and transparency in reporting. For further information on Nature Portfolio policies, see our Editorial Policies and the Editorial Policy Checklist.

Statistics

For all statistical analyses, confirm that the following items are present in the figure legend, table legend, main text, or Methods section.

n/a	Confirmed	
☐	☒	The exact sample size (*n*) for each experimental group/condition, given as a discrete number and unit of measurement
☐	☒	A statement on whether measurements were taken from distinct samples or whether the same sample was measured repeatedly
☐	☒	The statistical test(s) used AND whether they are one- or two-sided<br>*Only common tests should be described solely by name; describe more complex techniques in the Methods section.*
☐	☒	A description of all covariates tested
☐	☒	A description of any assumptions or corrections, such as tests of normality and adjustment for multiple comparisons
☐	☒	A full description of the statistical parameters including central tendency (e.g. means) or other basic estimates (e.g. regression coefficient) AND variation (e.g. standard deviation) or associated estimates of uncertainty (e.g. confidence intervals)
☐	☒	For null hypothesis testing, the test statistic (e.g. *F*, *t*, *r*) with confidence intervals, effect sizes, degrees of freedom and *P* value noted<br>*Give P values as exact values whenever suitable.*
☒	☐	For Bayesian analysis, information on the choice of priors and Markov chain Monte Carlo settings
☒	☐	For hierarchical and complex designs, identification of the appropriate level for tests and full reporting of outcomes
☐	☒	Estimates of effect sizes (e.g. Cohen's *d*, Pearson's *r*), indicating how they were calculated

Our web collection on statistics for biologists contains articles on many of the points above.

Software and code

Policy information about availability of computer code

Data collection	*Provide a description of all commercial, open source and custom code used to collect the data in this study, specifying the version used OR state that no software was used.*
Data analysis	Statistical analysis: R v. 4.0 (open source)<br>igraph R package (open source)<br>visNetwork R package (open source)<br>EPIC platform as described in Yeo et al. 2020, Nature Biotechnology (utilizing the Shiny package in R)<br>Prism version 8 (GraphPad Software)<br>SAS version 9.4 (SAS Institute Inc)<br>FlowJo 10.4.1 for Mac (Becton, Dickinson and Company, USA)

For manuscripts utilizing custom algorithms or software that are central to the research but not yet described in published literature, software must be made available to editors and reviewers. We strongly encourage code deposition in a community repository (e.g. GitHub). See the Nature Portfolio guidelines for submitting code & software for further information.

Data

Policy information about availability of data

All manuscripts must include a data availability statement. This statement should provide the following information, where applicable:

- Accession codes, unique identifiers, or web links for publicly available datasets
- A description of any restrictions on data availability
- For clinical datasets or third party data, please ensure that the statement adheres to our policy

Source data are provided with this paper.

Field-specific reporting

Please select the one below that is the best fit for your research. If you are not sure, read the appropriate sections before making your selection.

☒ Life sciences ☐ Behavioural & social sciences ☐ Ecological, evolutionary & environmental sciences

For a reference copy of the document with all sections, see nature.com/documents/nr-reporting-summary-flat.pdf

Life sciences study design

All studies must disclose on these points even when the disclosure is negative.

Sample size	Sample size was determined by study recruitment.
Data exclusions	Patient A08 was excluded from immunological analyses as they were lost to followup (rationale detailed in Methods). Additionally, samples with too few viable cells were excluded during QC steps of mass cytometry data analysis. Manual gating allowed for a greater number of samples to be included, but there were still some samples that had to be excluded due to low cell number. The marker 167Er-TCRa7.2 was excluded from clustering analyses in our routine quality control steps due to signal spillover from 168Er-IFNg.
Replication	Replication was not possible due to the restricted number of available samples. Reproducibility between mass cytometry runs was ensured with the use of a bridging control.
Randomization	Randomization was not performed for this study. The trial was designed as a dose-escalation trial. As described in the Methods section, we enrolled patients in cohorts of three for up to four dose levels (single subcutaneous administration of 1X109, 1X1010, 5X1010, 1X1011 viral particles) to establish the MTD. Patients in subsequent three cohorts were treated with multiple subcutaneous injections at the MTD, or if one is not established, the highest dose level stipulated in our protocol (i.e., 1X1011 viral particles). Patients in cohort 5 received inoculations on days 1 and 8 and patients in cohort 6 days received the vaccine on days 1, 8, and 22. If no dose-limiting toxicity (DLT) is observed in cohort 6, then a further six patients were enrolled into cohort 7 (the dose-expansion phase of the trial).
Blinding	PBMC samples were processed and acquired for mass cytometry blind. Samples were unblinded after de-barcoding and prior to statistical analysis.

Reporting for specific materials, systems and methods

We require information from authors about some types of materials, experimental systems and methods used in many studies. Here, indicate whether each material, system or method listed is relevant to your study. If you are not sure if a list item applies to your research, read the appropriate section before selecting a response.

Materials & experimental systems

n/a	Involved in the study	
☐	☒	Antibodies
☒	☐	Eukaryotic cell lines
☒	☐	Palaeontology and archaeology
☒	☐	Animals and other organisms
☐	☒	Human research participants
☐	☒	Clinical data
☒	☐	Dual use research of concern

Methods

n/a	Involved in the study	
☒	☐	ChIP-seq
☐	☒	Flow cytometry
☒	☐	MRI-based neuroimaging

Antibodies

Antibodies used	Antibodies used, their supplier, catalog number and clone name are listed in Supplementary Tables 5 and 6.
Validation	Performance of antibodies was checked using bivariate gating as in Yeo et al. 2020, Nature Biotechnology.

Human research participants

Policy information about studies involving human research participants

Population characteristics	Population characteristics of the cohort are listed in Supplementary Table 1 and detailed in the Study Protocol attached.
Recruitment	The primary physicians in consideration for the study referred patients for participation. There was no advertisement of the trial to the public. The trial was performed at the National Cancer Centre, Singapore, between September 2014 and November 2018.
Ethics oversight	All research in this open-label phase 1 trial (ClinicalTrials.gov identifier: NCT02140996) was approved by the SingHealth centralised institutional review board. Written informed consent was obtained from all patients before enrolment. All procedures involving human participants were carried out in accordance with the Declaration of Helsinki and principles of Good Clinical Practice.

Note that full information on the approval of the study protocol must also be provided in the manuscript.

Clinical data

Policy information about clinical studies

All manuscripts should comply with the ICMJEguidelines for publication of clinical research and a completedCONSORT checklist must be included with all submissions.

Clinical trial registration	NCT02140996
Study protocol	The Study Protocol is attached in the Supplementary Materials.
Data collection	The trial was performed at the National Cancer Centre, Singapore, between September 2014 and November 2018.
Outcomes	Part 1 of this trial comprised patients Cohort 1-4, where patients received a single subcutaneous administration of the Ad-sig-hMUC-1/ecdCD40L vector vaccine. The primary objective of part 1 was to determine the maximum tolerated dose (MTD of the Ad-sig-hMUC-1/ecdCD40L vector when administered once to each participating subject. In Part 2, containing cohorts 5 and 6, the primary objective was to test toxicity and efficacy of two successive administrations of the vaccine (seven days apart) in Cohort 5 and three successive administrations of the vector 7 and 14 days apart in cohort 6 (on Days 1, 8 and 22). In Part 3, the dose expansion phase (Cohort 7), patients will received 5 subcutaneous injections of the MUC-1 vaccine on Days 1, 8, 22, 52 and 82 in order to determine the safety and efficacy of 5 vaccinations. Outcomes were assessed by evaluation of DLT (see study protocol).

Flow Cytometry

Plots

Confirm that:

- [x] The axis labels state the marker and fluorochrome used (e.g. CD4-FITC).
- [x] The axis scales are clearly visible. Include numbers along axes only for bottom left plot of group (a 'group' is an analysis of identical markers).
- [x] All plots are contour plots with outliers or pseudocolor plots.
- [x] A numerical value for number of cells or percentage (with statistics) is provided.

Methodology

Sample preparation	Thawed PBMC were rested for 30 minutes at 37°C before being divided for further manipulation as previously described. To induce cytokine production, cells were stimulated with 150ng/mL phorbol 12-myristate 13 acetate (PMA) and 250ng/mL ionomycin (Sigma-Aldrich) for 4 hours, with Brefeldin A and monensin (eBioscience) added after the first hour of incubation. Cells were then stained with two separate panels of metal-conjugated antibodies as listed in Supplementary Tables 5 and 6. After staining and fixation with P1 or P2, cells were stored in 1% paraformaldehyde overnight or until acquisition.
Instrument	Helios mass cytometer (Fluidigm)

Software	CyTOF software version 6.7.1014. The Helios-generated output files were normalized using EQTM Four Element Calibration Beads.
Cell population abundance	Used without prior cell sorting
Gating strategy	Gating strategy provided in Supplementary Information

☒ Tick this box to confirm that a figure exemplifying the gating strategy is provided in the Supplementary Information.
